# DLK-1, SEK-3 and PMK-3 Are Required for the Life Extension Induced by Mitochondrial Bioenergetic Disruption in *C*. *elegans*

**DOI:** 10.1371/journal.pgen.1006133

**Published:** 2016-07-15

**Authors:** Erin Munkácsy, Maruf H. Khan, Rebecca K. Lane, Megan B. Borror, Jae H. Park, Alex F. Bokov, Alfred L. Fisher, Christopher D. Link, Shane L. Rea

**Affiliations:** 1 The Barshop Institute for Longevity and Aging Studies, University of Texas Health Science Center at San Antonio, San Antonio, Texas, United States of America; 2 Department of Cellular & Structural Biology, University of Texas Health Science Center at San Antonio, San Antonio, Texas, United States of America; 3 Department of Physiology, University of Texas Health Science Center at San Antonio, San Antonio, Texas, United States of America; 4 Department of Medicine (Division of Geriatrics, Gerontology, and Palliative Medicine), University of Texas Health Science Center at San Antonio, San Antonio, Texas, United States of America; 5 Department of Epidemiology and Biostatistics, University of Texas Health Science Center at San Antonio, San Antonio, Texas, United States of America; 6 Geriatric Research, Education and Clinical Center, South Texas VA Health Care System, San Antonio, Texas, United States of America; 7 Center for Healthy Aging, University of Texas Health Science Center at San Antonio, San Antonio, Texas, United States of America; 8 Institute for Behavioral Genetics & Department of Integrative Physiology, University of Colorado at Boulder, Boulder, Colorado, United States of America; Stanford University Medical Center, UNITED STATES

## Abstract

Mitochondrial dysfunction underlies numerous age-related pathologies. In an effort to uncover how the detrimental effects of mitochondrial dysfunction might be alleviated, we examined how the nematode *C*. *elegans* not only adapts to disruption of the mitochondrial electron transport chain, but in many instances responds with extended lifespan. Studies have shown various retrograde responses are activated in these animals, including the well-studied ATFS-1-dependent mitochondrial unfolded protein response (UPR^mt^). Such processes fall under the greater rubric of cellular surveillance mechanisms. Here we identify a novel p38 signaling cascade that is required to extend life when the mitochondrial electron transport chain is disrupted in worms, and which is blocked by disruption of the Mitochondrial-associated Degradation (MAD) pathway. This novel cascade is defined by DLK-1 (MAP3K), SEK-3 (MAP2K), PMK-3 (MAPK) and the reporter gene *Ptbb-6*::*GFP*. Inhibition of known mitochondrial retrograde responses does not alter induction of *Ptbb-6*::*GFP*, instead induction of this reporter often occurs in counterpoint to activation of SKN-1, which we show is under the control of ATFS-1. In those mitochondrial bioenergetic mutants which activate *Ptbb-6*::*GFP*, we find that *dlk-1*, *sek-3* and *pmk-3* are all required for their life extension.

## Author Summary

In humans, mitochondrial dysfunction contributes to numerous age-related diseases, and indeed even aging itself. Yet organisms also have an amazing capacity to compensate for mitochondrial impairment, paradoxically sometimes even living longer for it. This is exemplified in the roundworm *Caenorhabditis elegans*. In this study we examine how *C*. *elegans* with disrupted mitochondrial electron transport chains respond to such dysfunction and delineate a novel signaling cascade that is required for their life extension. Significantly, the components of this pathway are well-conserved in humans.

## Introduction

Once considered relatively rare, mitochondrial disorders are now recognized as one of the most common inherited human diseases [1]. Mitochondrial dysfunction is a causative factor in many of the major diseases that limit life-expectancy in humans [2] and is associated with chronic diseases such as type 2 diabetes [3], metabolic syndrome [4], Alzheimer’s disease [5, 6], Parkinson’s disease [7], depression [8], blindness [9] and even aging itself [10–13].

There is hope, however, for coping with, or even overcoming, some forms of mitochondrial dysfunction. In humans, diseases that affect the mitochondrial electron transport chain are pleiotropic and may take years to manifest. Some people remain asymptomatic [14], and there are even examples of spontaneous recovery [15]. This reflects complex interactions with other genes [16] and the environment [17], and suggests that cells are able to adapt to some level of mitochondrial impairment. Even more striking are those organisms that adapt to mitochondrial electron transport chain (ETC) disruption and actually have a longer lifespan as a result of it. This has been reported across phyla–including mice [18]ȁbut has been most extensively studied in the nematode *Caenorhabditis elegans* [19].

*C*. *elegans’* response to mitochondrial ETC dysfunction is threshold dependent; low levels produce no phenotype, moderate levels can result in increased lifespan, while severe disruption, as in humans, leads to overt pathology and shortened lifespan [20]. Intriguingly, research suggests that pathology resulting from severe mitochondrial dysfunction develops not as a direct consequence, but from the cell’s maladaptive response to the compromised mitochondria. For example, when the p53 homolog, *cep-1*, is knocked out, worms become long-lived when subjected to levels of mitochondrial disruption that would otherwise shorten lifespan [21]. This gives greater hope that we may be able to target and modulate such responses in humans.

The central role of mitochondria in the pathogenesis of multiple diseases is in part a consequence of their essential role in various cellular processes, including apoptotic signaling [22], ATP production [23], calcium sequestration [24], Fe-S cluster formation [25], immunity [26], nucleotide biosynthesis [27], oxidative stress signaling [28], stem cell maturation [29], steroid biosynthesis and xenobiotic detoxification [30]. The essential nature of mitochondria necessitates their functional status be closely monitored and it is now well established that signaling between the nucleus and mitochondria is bi-directional [31]. So-called retrograde response signaling originates from mitochondria and functions to orchestrate adaptive changes in nuclear gene expression to resolve or reduce mitochondrial stress. A variety of retrograde responses are known and are activated by an assortment of mitochondrial stressors, including depletion or mutation of mtDNA [32], reduced ETC activity [33], reduced mtDNA translation [34], oxidative stress [35], misfolded protein aggregation [36], altered mitochondrial turnover dynamics [37] and exposure to bacterial toxins [38].

Part of how organisms recognize a pathogen attack and activate an immune response is by monitoring their own core cellular functions, including cytosolic protein translation and mitochondrial function [38, 39]. Disruption of such processes is preemptively interpreted as evidence of a pathogen attack. This is a key adaptation for host organisms because the mechanisms of survival and reproduction of pathogens often remain critically dependent upon disrupting core cellular processes, even though pathogens may evolve to evade recognition by other forms of immune surveillance. As one example, many pathogens remain obliged to meet their requirement for iron by stealing it from their host’s mitochondria through use of siderophores [40]. Mitochondrial retrograde responses can be viewed therefore in a much broader sense as signaling elements of the cell surveillance system. The well-studied mitochondrial unfolded protein response (UPR^mt^) is one type of retrograde response and, in *C*. *elegans*, it is activated by a number of bacteria native to its habitat. Interestingly, the UPR^mt^ can also be suppressed by alternate branches of the cell surveillance system when other responses are deemed more urgent [41].

The UPR^mt^ in worms has been well characterized by the Ron and Haynes labs [42] and this process is critical for both development in the face of mitochondrial disruption [43] and for resistance to infection [39]. Two studies utilizing RNAi knockdown of *ubl-5* –an important factor mediating the UPR^mt^ response [44]–suggested that the UPR^mt^ may be specifically required for life extension in response to mitochondrial dysfunction [18, 45]. However, *ubl-5* may have a constitutive role in mitochondrial homeostasis beyond UPR^mt^ induction, making the UPR^mt^-specific transcription factor, *atfs-1* [43], a better candidate to test the involvement of UPR^mt^ in longevity [46]. Contrary to expectation, not only does constitutively active ATFS-1 fail to extend lifespan [47], removal of *atfs-1* by RNAi or mutation does not prevent life extension following mitochondrial disruption by *isp-1(qm150)* or *cco-1* RNAi [46]. These results suggest that activation of the UPR^mt^ may not produce the life extension observed upon mitochondrial dysfunction. Similarly, a recent study on the proteomes of several long-lived mouse models found that longevity correlated with decreased expression of multiple subunits of complexes I, III, IV and V and that this was not accompanied by any activation of the UPR^mt^ [48]. Thus we set out to find other signaling pathways that are triggered independently of *atfs-1* in response to mitochondrial dysfunction, and which might instead be required for life extension.

## Results

### *tbb-6* Marks a Novel Signaling Response to Mitochondrial Dysfunction

**Fig 1:**
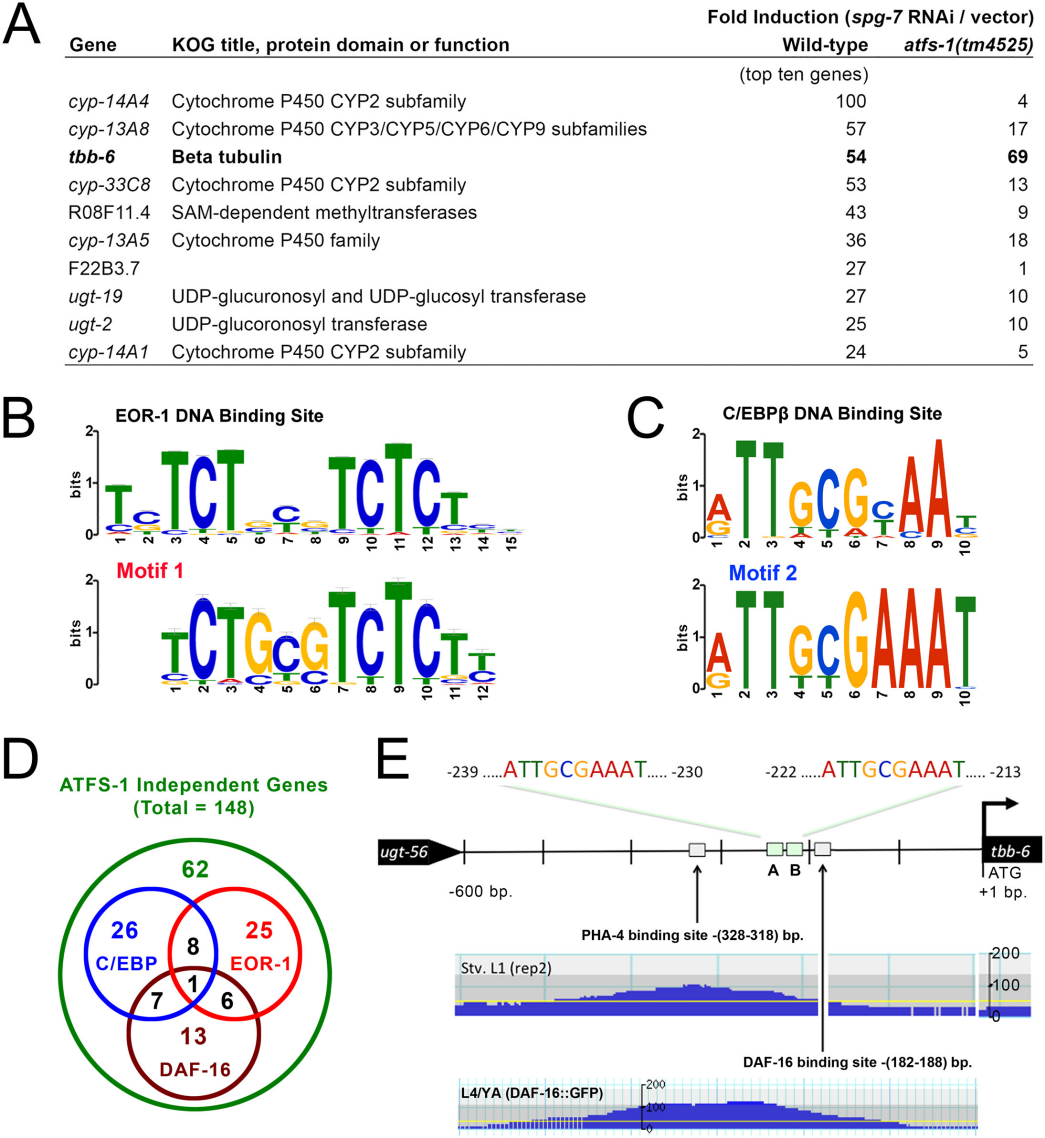
Evidence for a novel signaling pathway activated subsequent to mitochondrial disruption. (**A**) Among the ten most highly upregulated genes activated following mitochondrial disruption by *spg-7* RNAi, *tbb-6* alone does not require *atfs-1* for its induction (microarray data from GEO dataset GSE38196). See also S1 Table. (**B**) 40 of the 148 *atfs-1* independent genes activated following *spg-7* disruption contain a predicted EOR-1 binding motif (shown in LOGO form aligned against the consensus EOR-1 site which was identified through the *C*. *elegans* ModENCODE project (*top panel*)). (**C**) 42 of the 148 *atfs-1* independent genes activated following *spg-7* disruption contain a C/EBP-like promoter motif (shown in LOGO form aligned against the promoter motif bound by human C/EBPβ (*top panel*)). (**D**) Venn diagram illustrating the degree of overlap between groups of *atfs-1* independent genes that contain C/EBPβ -like, EOR-1 or DAF-16 promoter elements. (**E**) Promoter region of *tbb-6*: Sites A and B match the human C/EBPβ consensus motif shown in panel (C). ChiP-Seq data from the *C*. *elegans* ModENCODE project [52], reveals a functional DAF-16 binding site, as well as a functional PHA-4 binding site [Stv. L1(rep 2)–starved L1 larvae, 2^nd^ replicate sample set, L4/YA—larval stage 4/young adult].

To identify genes in *C*. *elegans* that are upregulated independently of ATFS-1 following mitochondrial ETC disruption, we utilized previously published microarray data [43]. We identified 148 genes upregulated more than two-fold in wild-type worms (N2 Bristol) treated with RNAi targeting the mitochondrial metalloprotease *spg-7* and which remained elevated in mutant *atfs-1(tm4525)* worms following the same RNAi treatment (S1 Table). Of these genes, the one showing greatest induction upon mitochondrial disruption was the uncharacterized β-tubulin, *tbb-6*. It was upregulated more than fifty-fold in wild-type animals, and nearly seventy-fold in *atfs-1(tm4525)* worms. Indeed, *tbb-6* was among the ten most highly upregulated of all genes following s*pg-7* RNAi treatment and, of these ten, the only one that did not require *atfs-1* for its induction (Fig 1A). Promoter analysis of the 148 *atfs-1* independent genes identified five motifs that were significantly over-represented: Three motifs were restricted to six small heat shock proteins and all were related to the well-characterized heat shock regulatory element [49]. Forty genes (27%) contained one or more EOR-1 binding motifs (significant at a *p-value* of 2.1e-43) (Fig 1B and S1 Table), while forty-two genes (28%) contained one or more CCAAT/enhancer binding protein (C/EBP)-like motifs (significant at a *p-value* of 3.2e-31) (Fig 1C and S1 Table). Interestingly, the two groups of genes containing the latter two motifs were largely independent of each other (Fig 1D and S1 Table). DAF-16/FOXO is a transcription factor best known for its role in life extension following inhibition of the insulin/IGF-1-like signaling pathway in worms [50] but has repeatedly been shown to be uninvolved in life extension following mitochondrial disruption (reviewed in [19]). Recent studies have shown that half of all promoters bound by DAF-16 also contain one or more EOR-1 binding motifs [51], yet of the forty genes that we identified with EOR-1 binding motifs, only seven also contained a DAF-16 binding motif (Fig 1D and S1 Table). DAF-16 binding sites were not significantly over represented in our sample set beyond expectation, even though there were 27 genes containing one or more matches to the DAF-16 binding site consensus (Fig 1D). The promoter of *tbb-6* contains two C/EBP motifs, as well as PHA-4 and DAF-16 binding sites (Fig 1E and S1 Table). Collectively, our data hint at the presence of one or more unexplored signaling pathways that are activated independently of the ATFS-1 dependent UPR^mt^ pathway, and which function downstream of mitochondrial disruption to coordinately modulate the expression of multiple genes. Given the extent to which *tbb-6* is upregulated upon mitochondrial disruption (~70 fold), we reasoned that *tbb-6* expression would serve as a useful marker for a potentially unexplored mitochondrial retrograde response that controls lifespan.

### A *tbb-6* Transcriptional Reporter Is Induced following Mitochondrial ETC Disruption

**Fig 2:**
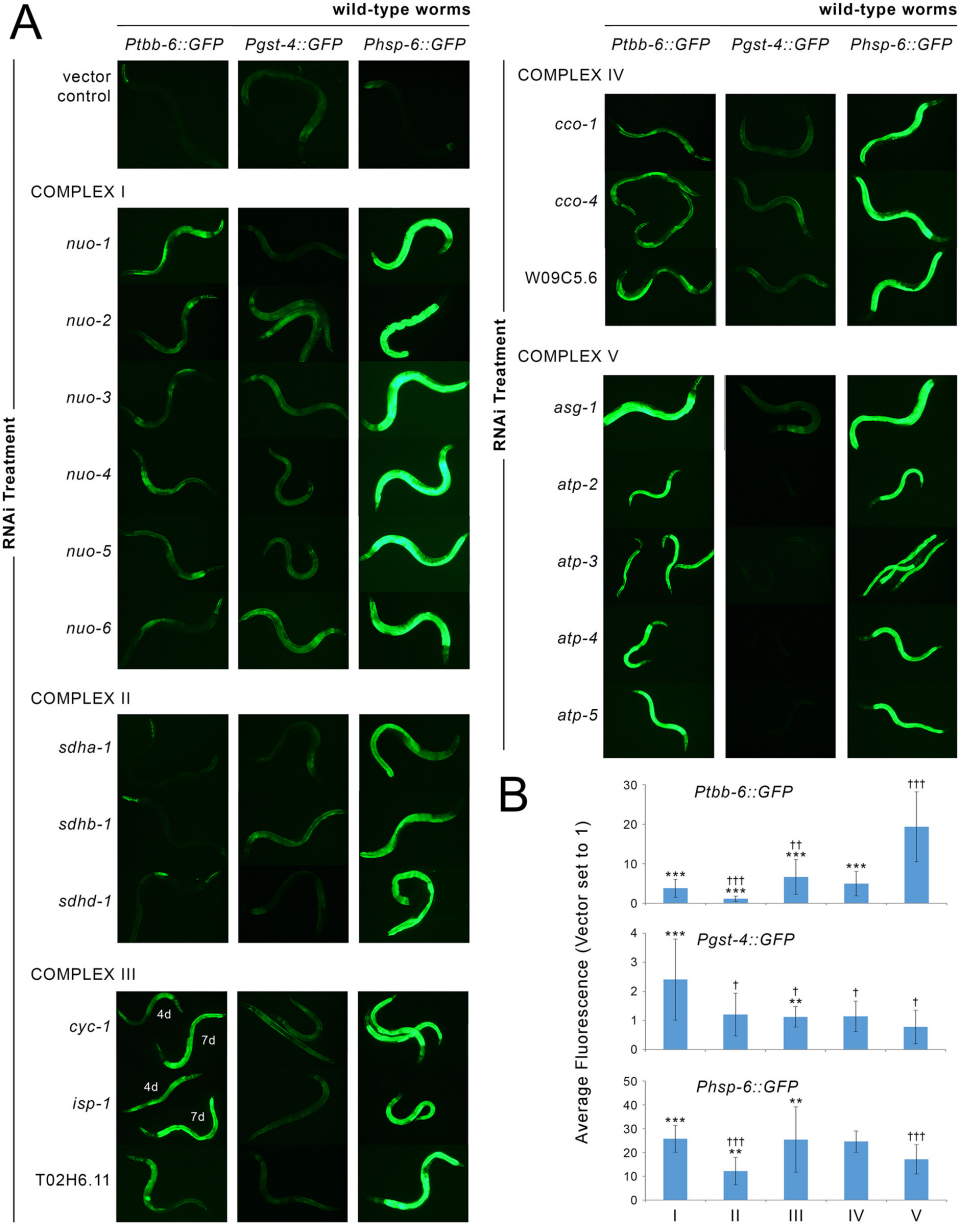
*Ptbb-6*::*GFP* reporter activation following mitochondrial ETC disruption. (**A**) RNAi-mediated knockdown of mitochondrial respiratory chain subunits differentially induces *Ptbb-6*::*GFP* reporter expression relative to *Pgst-4*::*GFP* and *Phsp-6*::*GFP*. Shown are representative fluorescence images from a selection of subunits targeted in each complex. Quantified data of multiple replicates for all tested subunits is provided in S1–S3 Figs. (**B**) Mean change in GFP reporter fluorescence (+/-SD) when the effect of RNAi treatments targeting subunits from each ETC complex are averaged. Two statistical comparisons are shown (Student’s t-test with Bonferroni correction applied for multiple comparisons): Asterisks indicate ETC complex disruptions which, on average, differ significantly in GFP fluorescence relative to knockdown of complex V subunits. Double daggers indicate ETC complex disruptions which, on average, differ significantly in GFP fluorescence relative to knockdown of complex I subunits. (*/†, p<0.01; **/††, p<0.001; ***/ǂǂǂ, p<0.00001) For complex III subunits *cyc-1* and *isp-1*, 4d and 7d refer to 4 day—and 7-day old worms. For comparisons relative to empty vector see S4 Fig.

We constructed a *Ptbb-6*::*GFP* transcriptional reporter strain and observed background expression in the pharynx, which is consistent with the presence of a PHA-4 binding site in the *tbb-6* promoter region (Fig 1E). There was no expression of GFP anywhere else in these worms (see vector control in Fig 2A). We tested whether the *Ptbb-6*::*GFP* reporter, like the UPR^mt^, could be induced upon various RNAi-mediated disruptions to the mitochondrial ETC and related proteins. In all instances where the reporter was activated in adult worms, we observed strongest expression in the intestine (Fig 2A). We also detected faint neuronal expression on some occasions, and during the L4 larval stage *Ptbb-6*::*GFP* was often transiently but strongly expressed in the hypodermis. In adult worms, depending upon which respiratory complex was affected, the level of *Ptbb-6*::*GFP* expression varied greatly. On average, RNAi knockdown of subunits of complex V led to the highest *Ptbb-6*::*GFP* induction. Expression was lower, but still well above background, following knockdown of complex I, III or IV subunits (Figs 2, S1 and S4 and S2 Table). In contrast, using these same RNAi treatments, *Pgst-4*::*GFP* expression was most strongly induced upon disruption of complex I (Figs 2 and S2). This reporter is controlled by the oxidative stress sensitive SKN-1/NRF2 transcription factor. With few exceptions, the UPR^mt^-specific reporter *Phsp-6*::*GFP* was robustly induced when any subunit of the electron transport chain was disrupted (Figs 2 and S3). Removing paralogous subunits from our analysis did not change our overall conclusions (S4 Fig). Finally, we also observed induction of *Ptbb-6*::*GFP* expression using four additional RNAi clones that disrupt mitochondrial function and can increase lifespan–*hsp-6*, *mrpl-47*, *mrps-5* and F13G3.7 (orthologous to human SLC25A44) (S5 Fig).

**Fig 3:**
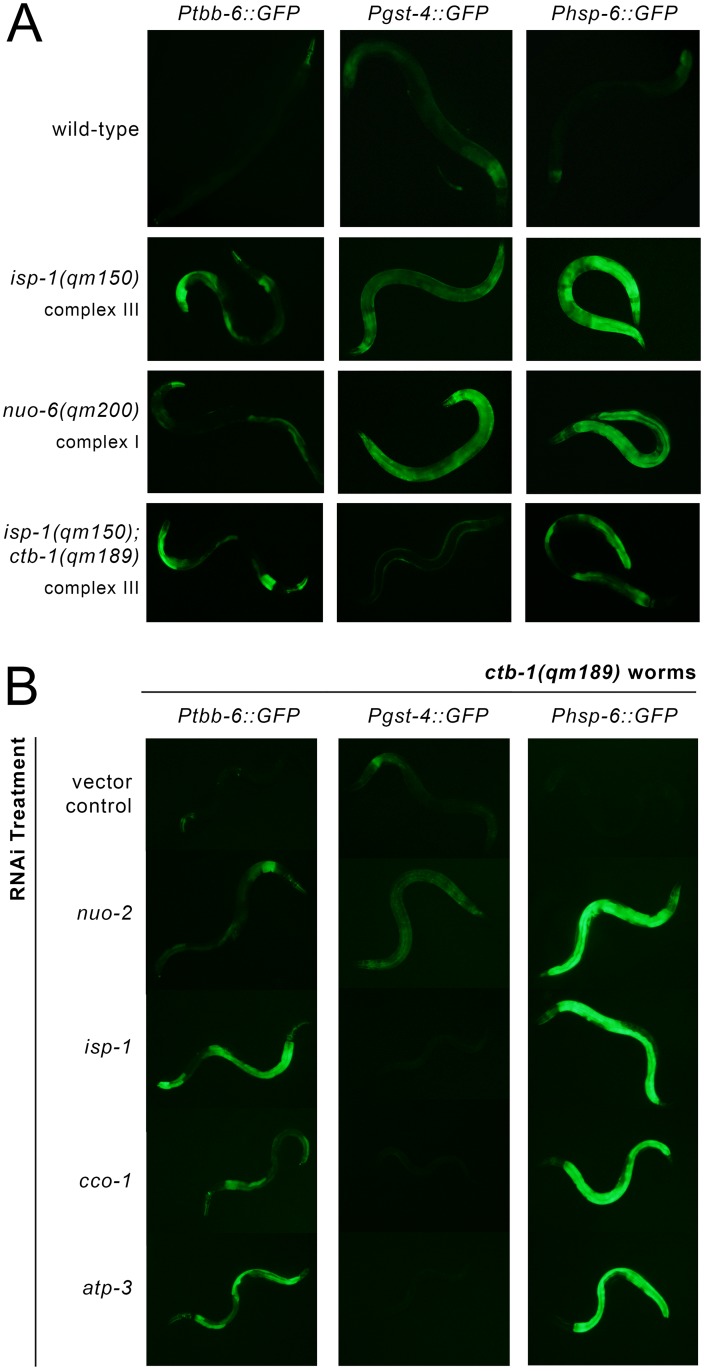
*Ptbb-6*::*GFP* reporter expression defines a UPR^mt^ independent pathway. (**A**) *Ptbb-6*::*GFP* is less strongly induced by mutation of ETC subunits than by RNAi knockdown, whereas *Pgst-4*::*GFP* expression displays an opposite pattern. (**B**) Treatment of *ctb-1(qm189)* mutants with RNAi targeting complexes I, III, IV or V [*nuo-2*, *isp-1*, *cco-1* and one-tenth strength *atp-3*, respectively], underscores the reciprocal relationship between *Ptbb-6*::*GFP* and *Pgst-4*::*GFP* reporter expression. Quantification data for both (A) and (B) is provided in S6 Fig.

Previous work has suggested that mitochondrial dysfunction resulting from genetic mutations that disrupt subunits of the ETC may invoke retrograde responses that are fundamentally different from those activated following RNAi-knockdown of the same ETC subunits [53]. We crossed our three transcriptional reporters into *isp-1(qm150)* [54] and *nuo-6(qm200)* [53] worms to see if their mutations (disrupting complexes III and I, respectively), would also lead to *tbb-6* induction. In line with our RNAi data, *Ptbb-6*::*GFP* was induced in *isp-1(qm150)* worms, but markedly less so in *nuo-6(qm200)* animals. Interestingly, *Pgst-4*::*GFP* exhibited the reciprocal expression phenotype, while both *isp-1(qm150)* and *nuo-6(qm200)* worms strongly induced the *Phsp-6*::*GFP* reporter (Fig 3A). *ctb-1(qm189)* is a mitochondrial DNA mutation which alters cytochrome b of complex III. This mutation attenuates the slow development of *isp-1(qm150)* worms but not their extended lifespan [54]. By itself, the *ctb-1(qm189)* mutation reduces complex III activity by up to 50% compared to wild-type animals [55]. Crossing our three transcriptional reporters into both *ctb-1(qm189)* and *isp-1(qm150); ctb-1(qm189)* genetic backgrounds, we observed that *Ptbb-6*::*GFP* and *Phsp-6*::*GFP* were each induced in *isp-1(qm150); ctb-1(qm189)* double mutants (but less so than in *isp-1(qm150)* animals), while there was no expression at all of the *Pgst-4*::*GFP* reporter (Fig 3A). *ctb-1(qm189)* mutants, instead, showed the reciprocal pattern of reporter protein induction (Figs 3B and S6A). These findings are intriguing because within *isp-1(qm150); ctb-1(qm189)* mutants the *ctb-1(qm189)* mutation increases the activity of complex I specifically within supercomplex assemblies [55]. A reciprocal relationship between *Ptbb-6*::*GFP* and *Pgst-4*::*GFP* reporter expression was further underscored when *ctb-1(qm189)* worms were exposed to RNAi targeting different subunits of the ETC (Figs 3B and S6B). Taken together, these data show that *Ptbb-6*::*GFP* is broadly induced by mitochondrial disruption and that its expression appears independent of UPR^mt^ activation. Intriguingly, *Ptbb-6*::*GFP* induction exhibits a strong complementarity to SKN-1 activation (Figs 2 and 3). These findings suggest that *tbb-6* could indeed reflect activation of a novel retrograde response.

### *tbb-6* Activation Does Not Depend on DAF-16, SKN-1 nor ATFS-1

It has been shown repeatedly that DAF-16 is unnecessary for the life extension that follows mitochondrial ETC disruption in *C*. *elegans* [12, 13, 21, 45, 54, 56, 57]. Since the *tbb-6* promoter contains a DAF-16 binding element, we nonetheless tested the role of this transcription factor in *tbb-6* promoter activation. Knock-down of *daf-16* by RNAi in *isp-1(qm150); Ptbb-6*::*GFP* worms failed to block reporter gene induction (Table 1).

**Table 1:** Targeted screen for factors regulating *Ptbb-6*::*GFP* expression and larval development in *isp-1(qm150)* worms (quantified relative to vector-treated animals).

RNAi	Gene Name	Gene Function	*Ptbb-6*::*GFP*	Development	Reference
***MAPKs***					
B0478.1	*jnk-1*	Jun-N-terminal MAPK (stress response)	**+**	no effect	
T07A9.3	*kgb-1*	Jun-N-terminal MAPK (stress response)	no effect	no effect	
ZC416.4	*kgb-2*	Jun-N-terminal MAPK (stress response)	no effect	no effect	
C49C3.10		Jun-N-terminal MAPK (stress response)	no effect	no effect	
Y51B9A.9		Jun-N-terminal MAPK (stress response)	no effect	no effect	
B0218.3	*pmk-1*	p38 MAPK (stress response)	no effect	no effect	
F42G8.3	*pmk-2*	p38 MAPK (stress response)	no effect	no effect	
F42G8.4	*pmk-3*	p38 MAPK (stress response)	---	no effect	
F43C1.2	*mpk-1*	ERK MAPK (growth response factor)	no effect	no effect	
W06B3.2	*sma-5*	ERK MAPK (development)	+++	**delay/arrest**	
W06F12.1	*lit-1*	nmo MAPK (development and inflammation)	no effect	no effect	
C04G6.1	*mpk-2*	MAPK	no effect	no effect	
C05D10.2		MAPK	-	no effect	
F09C12.2		related to MAPK	**-**	no effect	
***MAP2Ks***					
F35C8.3	*jkk-1*	MAPKK	**-**	no effect	
K08A8.1	*mek-1*	MAPKK	no effect	no effect	
Y54E10BL.6	*mek-2*	MAPKK	variable	no effect	
F42G10.2	*mkk-4*	MAPKK	+	no effect	
R03G5.2	*sek-1*	MAPKK	**--**	no effect	
ZC449.3	*sek-3*	MAPKK	**---**	no effect	
F35C8.2	*sek-4*	MAPKK	no effect	no effect	
F35C8.1	*sek-5*	MAPKK	no effect	no effect	
VZC374L.1	*sek-6*	MAPKK	no effect	no effect	
E02D9.1		MAPKK	no effect	no effect	
***MAP3Ks***					
F29C4.1	*daf-1*	MAPKKK	no effect	no effect	
C05D2.1	*daf-4*	MAPKKK	no effect	no effect	
F33E2.2	*dlk-1*	MAPKKK	--	no effect	
F13B9.5	*ksr-1*	MAPKKK	no effect	no effect	
F58D5.4	*ksr-2*	MAPKKK	no effect	no effect	
K11D12.10	*mlk-1*	MAPKKK	no effect	no effect	
F52F12.3	*mom-4*	MAPKKK	no effect	no effect	
B0414.7	*mtk-1*	MAPKKK	no effect	no effect	
F59A6.1	*nsy-1*	MAPKKK	no effect	no effect	
K09B11.1	*pik-1*	MAPKKK	no effect	no effect	
C24A1.3		MAPKKK	no effect	no effect	
Y105C5A.x		MAPKKK	no effect	no effect	
***Dual-Specificity Phosphatases***					
F08B1.1	*vhp-1*	dual-specificity MAPK phosphatase	+++	**arrested**	[61]
C04F12.8		potential dual-specificity MAPK phosphatase	no effect	no effect	
C24F3.2		potential dual-specificity MAPK phosphatase	no effect	no effect	
F13D11.3		potential dual-specificity MAPK phosphatase	no effect	no effect	
F28C6.8		potential dual-specificity MAPK phosphatase	no effect	no effect	
Y54F10BM.13		potential dual-specificity MAPK phosphatase	+	**delayed**	
ZK757.2		potential dual-specificity MAPK phosphatase	no effect	no effect	
***PMK-3 Signaling Pathway Interactors***					
D1005.3	*cebp-1*	bZIP TF; CCAAT-enhancer binding protein	no effect	no effect	[62]
C44C8.6	*mak-2*	MAP kinase activated protein kinase	+	**delayed**	[63]
F26H9.6*	*rab-5*	RAB5 GTPase ortholog	**+**	**arrested**	[64]
C01B7.6*	*rpm-1*	E3 ubiquitin ligase	**-**	no effect	[65]
F26H9.7	*uev-3*	ubiquitin-conjugating enzyme (E2) variant	**--**	no effect	[63]
***Transcription Factors modulating Mit Mutant Lifespan***					
C25A1.11	*aha-1*	AHA-1 interacts with AHR-1 and HIF-1 *in vitro*	no effect	**delayed**	[33]
ZC64.3	*ceh-18*	POU-class homeodomain transcription factor	+	**asynchronous**	[33]
ZK652.5*	*ceh-23*	homeodomain transcription factor	no effect	no effect	[66]
F52B5.5	*cep-1*	ortholog of human tumor suppressor p53	no effect	no effect	[21]
F38A6.3	*hif-1*	hypoxia-induced transcription factor	no effect	no effect	[67]
W02C12.3*	*hlh-30*	bHLH TF; lipid metabolism	no effect	**delayed**	[68]
T24H10.7	*jun-1*	bZIP TF; development	+	no effect	[33]
F16H9.2	*nhr-27*	nuclear hormone receptor transcription factor	no effect	no effect	[33]
K10C3.6	*nhr-49*	NHR transcription factor; lipid metabolism	no effect	**asynchronous**	[33]
R119.6	*taf-4*	TFIID transcription factor	+++	**delayed**	[33]
***Immune Response***					
C33D3.1*	*elt-2*	GATA-type TF; intestinal immunity	no effect	**delay/arrest**	[69]
C50H2.1	*fshr-1*	neuropeptide receptor	no effect	no effect	[70]
Y53C10A.12	*hsf-1*	heat-shock TF; stress and immune response	++	not tested	[71]
K02F3.4	*zip-2*	immune response	no effect	no effect	[72]
***spg-7 RNAi Induced***					
F45E4.1*	*arf-1*.*1*	ADP-ribosylation factor	no effect	no effect	[43]
ZC376.7	*atfs-1*	mitochondrial unfolded protein	+++	**delay/arrest**	[43]
K01D12.11*	*cdr-4*	cadmium responsive	**+**	no effect	[43]
F52E1.13*	*lmd-3*	oxidative resistance	**+**	no effect	[43]
F40F8.7	*pqm-1*	paraquat responsive	**+**	no effect	[43]
T19E7.2	*skn-1*	development; oxidative stress response	**+**	no effect	[43]
F47H4.10*	*skr-5*	homolog of Skp1 in *S*. *cerevisiae*	no effect	no effect	[43]
T16G1.4*		uncharacterized	+	no effect	[43]
***Cytoprotection***					
F57H12.1	*arf-3*	ADP-ribosylation factor	+++	no effect	[60]
F56A8.6*	*cpf-2*	mRNA cleavage	**+++**	**delayed**	[60]
F09G2.4*	*cpsf-2*	cleavage and polyadenylation specificity factor	**+**	**delayed**	[60]
D2045.6	*cul-1*	cullin; development	**+++**	**delayed**	[60]
R13H8.1	*daf-16*	forkhead box O (FOXO) transcription factor	no effect	no effect	[60]
C26C6.5*	*dcp-66*	ortholog of NuRD component p66	no effect	no effect	[60]
F47A4.2	*dpy-22*	mediator protein subunit	-	no effect	[60]
C33D3.1*	*elt-2*	GATA-type TF; intestinal immunity	no effect	**delay/arrest**	[60]
H13N06.3*	*gob-1*	trehalose-6-phosphatase	variable	**delayed**	[60]
C53A5.3*	*hda-1*	histone deacetylase	-	no effect	[60]
F25B4.6	*hmgs-1*	HMG-CoA synthase	**+++**	**delay/arrest**	[60]
F32E10.4*	*ima-3*	importin alpha nuclear transport factor	**++**	**delayed**	[60]
C41C4.4*	*ire-1*	ER unfolded protein response (UPR)	**+**	**delayed**	[60]
M7.1	*let-70*	E2 ubiquitin conjugating enzyme	**---**	**delay/arrest**	[60]
F38H4.9*	*let-92*	catalytic subunit of protein phosphatase 2A	**---**	**delay/arrest**	[60]
T27C4.4*	*lin-40*	component of NuRD complex	**--**	**delayed**	[60]
C25H3.6*	*mdt-26*	mediator; development	**+**	no effect	[60]
ZC581.1*	*nekl-2*	serine threonine protein kinase	**-**	**arrested**	[60]
T23H2.5*	*rab-10*	RAB-like GTPase	**+++**	no effect	[60]
C35C5.1*	*sdc-2*	regulates X transcription	no effect	**delayed**	[60]
F46A9.5*	*skr-1*	ubiquitin ligase complex component	no effect	no effect	[60]
C06A8.2*	*snpc-1*.*1*	small nuclear RNA activating complex	--	**delayed**	[60]
C23H3.4	*sptl-1*	serine palmitoyltransferase; development	**+**	**arrested**	[60]
F19B6.2	*ufd-1*	ubiquitin selection chaperone	**--**	**delay/arrest**	[60]
C46C2.1*	*wnk-1*	WNK-type protein kinase homolog	no effect	no effect	[60]
F53F4.11*		an ortholog of human RSL1D1	no effect	**delayed**	[60]
***Surveillance***					
F40F12.7	*cbp-3*	CREB binding protein	---	**arrested**	[26]
F31E3.1	*ceh-20*	homeodomain transcription factor	**-**	no effect	[26]
Y47G6A.23	*lpd-3*	lipid metabolism	variable	no effect	[26]
R05D11.3	*ran-4*	nuclear transport factor; development	variable	**delayed**	[26]
Y54E10BR.5		signal peptidase complex subunit	no effect	no effect	[26]
**Mitochondria-associated degradation (MAD)**					
C06A1.1	*cdc-48*.*1*	AAA-ATPase	**-**	**arrested**	
C41C4.8	*cdc-48*.*2*	AAA-ATPase	**-**	**arrested**	
F59E12.5	*npl-4*.*2*	ubiquitin selection chaperone	--	**arrested**	
F19B6.2	*ufd-1*	ubiquitin selection chaperone	--	**delay/arrest**	[60]
K06H7.3	*vms-1*	VCP/Cdc48-associated (controversial role)	no effect	no effect	[73–75]
***Other***					
Y116A8C.12*	*arf-6*	ADP-ribosylation factor	-	no effect	
C06A1.1	*cdc-48*.*1*	ubiquitin selection chaperone; ERAD	**-**	**arrested**	
C41C4.8	*cdc-48*.*2*	ubiquitin selection chaperone; ERAD	**-**	**arrested**	
C35D10.9	*ced-4*	programmed cell death	no effect	no effect	[76]
F56D2.7	*ced-6*	cell-corpse engulfment during apoptosis	no effect	no effect	
R13H8.1	*daf-16*	forkhead box O (FOXO) transcription factor	no effect	no effect	
C26D10.5	*eff-1*	involved in cell fusions	no effect	no effect	
F52E1.7	*hsp-17*	heat-shock protein chaperone	no effect	no effect	
C09H6.2*	*lin-10*	required for polarized protein localization	+	no effect	
F59E12.5	*npl-4*.*2*	ER-associated protein degradation (ERAD)	**-**	**arrested**	
F55B12.5	*nrf-5*	lipid-binding transportation protein	no effect	no effect	
F29B9.4	*psr-1*	apoptotic pathway	no effect	no effect	
C03C10.4	*rei-1*	RAB-11 GEF activity	**+++**	no effect	[77]
F10D11.1	*sod-2*	mitochondrial superoxide dismutase	-	no effect	[78]
C44H4.5*	*tap-1*	TGF-beta activated kinase	no effect	no effect	
F42D1.2	*tatn-1*	tyrosine amino transferase	no effect	no effect	
T04H1.9	*tbb-6*	beta-tubulin	no effect	no effect	[43]
R13F6.4	*ten-1*	teneurin	--	no effect	
ZK524.2	*unc-13*	regulator of neurotransmitter release	**+**	no effect	[79]
K06H7.3	*vms-1*	VCP/Cdc48-associated mito stress responsive	no effect	no effect	[73]
D2030.9	*wdr-23*	negative regulator of SKN-1	-	**delayed**	[80]
T20F10.1	*wts-1*	integrity of apical intestinal membrane	no effect	no effect	
C03C10.4		mitochondrial ribosome interacting protein	+++	no effect	

* RNAi clone was sequence not verified

**Fig 4:**
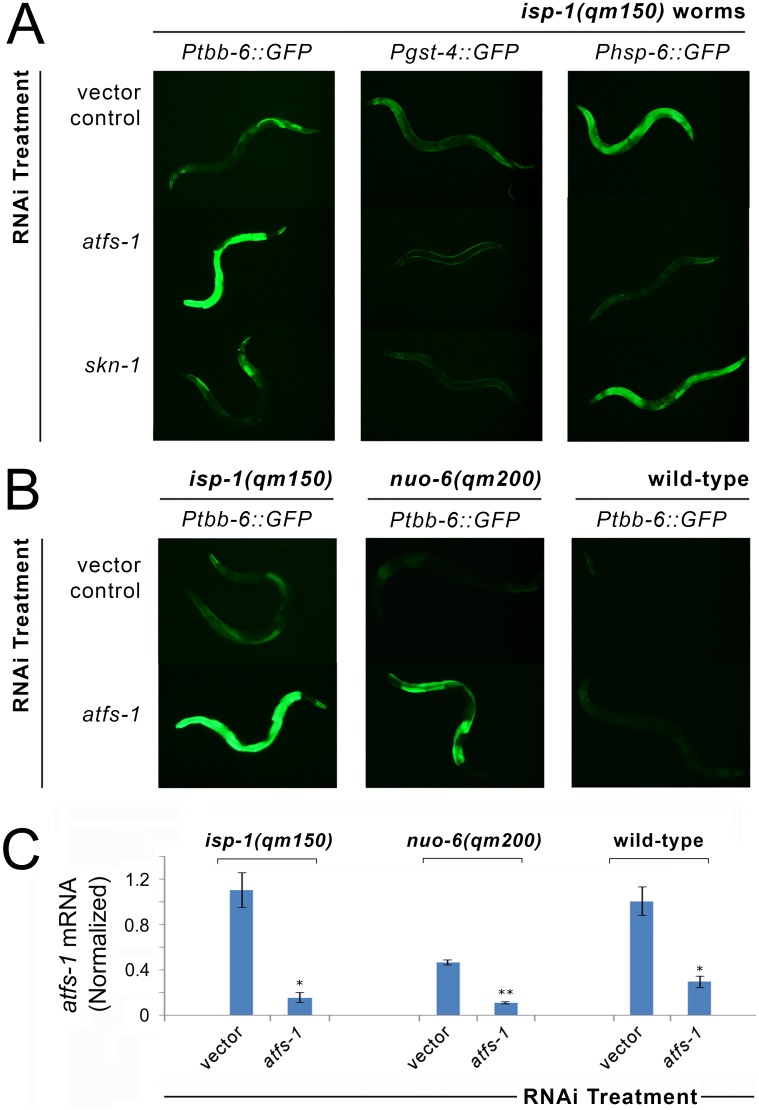
*Ptbb-6*::*GFP* reporter expression defines a UPR^mt^ independent pathway. (**A**) RNAi knockdown of *atfs-1* blocks *Phsp-6*::*GFP* expression, as reported [43], but dramatically further upregulates *Ptbb-6*::*GFP* in both *isp-1(qm150)* and *nuo-6(qm200)* worms. Surprisingly, *atfs-1* RNAi also turned off *Pgst-4*::*GFP*. RNAi knockdown of *skn-1* in both *isp-1(qm150)* and *nuo-6(qm200)* worms (data for latter worms is also provided in S7 Fig), turns off *Pgst-4*::*GFP*, as reported [59], but has no effect on *Ptbb-6*::*GFP* (and *Phsp-6*::*GFP)* expression. (**B**) Upregulation of *Ptbb-6*::*GFP* following *atfs-1* removal is only observed in the context of ETC dysfunction. (**C**) Quantitative of *atfs-1* mRNA in worms of panel (B). Bars represent mean (+/- SD); *n* = 3 biological replicates/condition. Asterisks indicate significant knockdown of *atfs-1* mRNA on *atfs-1* RNAi relative to vector (Student’s t-test, *p<0.001, **p<0.0001).

In worms, ATFS-1 is the master transcriptional regulator of the UPR^mt^, while SKN-1 is the key NRF-2 like transcription factor that responds to oxidative- and xenobiotic stresses [58]. The activation of both proteins has been reported in Mit mutants [20, 43]. As expected from our analysis of the *spg-7* microarray data, ATFS-1 is not required for *Ptbb-6*::*GFP* activation. In Fig 4A, we show that removal of *atfs-1* by RNAi completely blocked both *Phsp-6*::*GFP* [43] and *Pgst-4*::*GFP* induction in *isp-1(qm150)* Mit mutants, while *Ptbb-6*::GFP was not reduced. The dependency of SKN-1 activation on ATFS-1 following mitochondrial dysfunction has not been previously reported. Intriguingly, our data show that not only does SKN-1 sit downstream of ATFS-1, but it may also have a role in activation of downstream UPR^mt^ components; treating *isp-1(qm150)* and *nuo-6(qm200)* worms with *skn-1* RNAi completely blocked *Pgst-4*::*GFP* expression but also mildly, but significantly, attenuated *Phsp-6*::*GFP* induction (Figs 4A and S7A and S7B). In contrast to the other two reporters, *Ptbb-6*::GFP induction by *isp-1(qm150)* was not only undiminished by *atfs-1* RNAi, it was markedly further activated (see Fig A in S7 Fig for quantitation). Surprisingly, in *nuo-6(qm200)* worms, which minimally induce *Ptbb-6*::GFP, we also observed hyperactivation of *Ptbb-6*::*GFP* following *atfs-1* removal (**Fig B in** S7 Fig, quantification in **Fig C in** S7 Fig). Furthermore, our data reveal that *atfs-1* RNAi specifically induces *Ptbb-6*::*GFP* expression in the context of mitochondrial dysfunction, because the reporter remained unchanged when wild type worms were treated with the same *atfs-1* RNAi (Fig 4B). This difference in phenotype was unlikely to be due to differences in the efficacy of RNAi knockdown between the strains (Fig 4C). Thus *tbb-6* is not only *daf-16*, *atfs-1* and *skn-1* independent, but it is activated complementary to UPR^mt^ and oxidative stress signaling.

### *tbb-6* Marks a New Branch of the Cell Surveillance System

**Fig 5:**
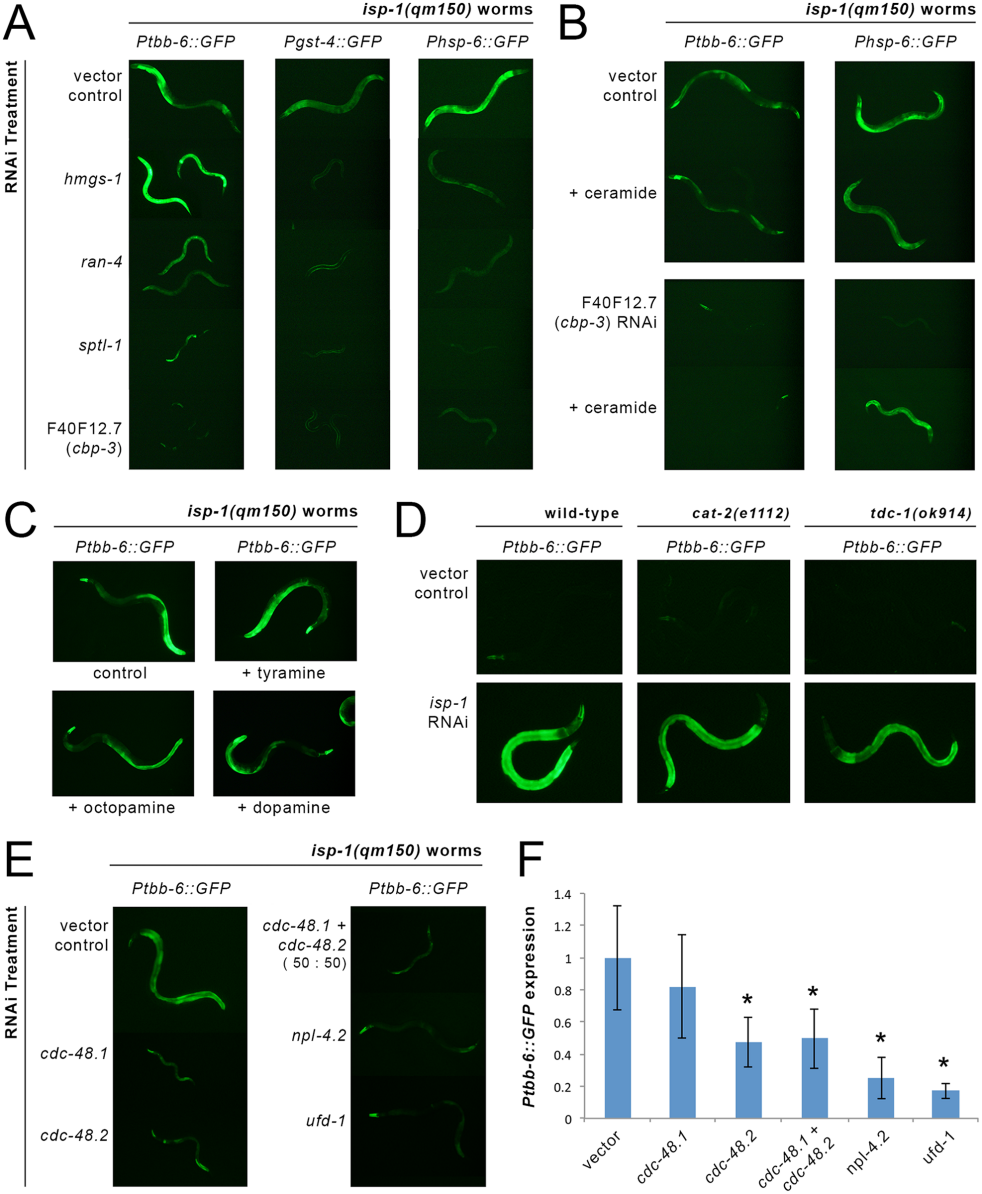
*Ptbb-6*::*GFP* marks a new cell surveillance pathway. (**A, B**) Among genes known to function epistatically to *atfs-1* in its role in activating the UPR^mt^ [26, 60], only F40F12.7/ *cbp-3* is also required for *Ptbb-6*::*GFP* expression (A). The role of *cbp-3* in the *Ptbb-6*::*GFP* pathway is distinct from its role in the UPR^mt^, since ceramide addition only replaces the requirement for *cbp-3* in UPR^mt^ activation (B). (**C, D**) Monoamine neurotransmission and neuromodulation are dispensable for *Ptbb-6*::*GFP* activation. Neither dietary supplementation of L-tyramine, octopamine or dopamine (C), nor genetic inactivation of catecholamine synthesis (D), alters *Ptbb-6*::*GFP* activation following mitochondrial ETC disruption. (**E, F**) RNAi-mediated inhibition of core MAD pathway genes strongly inhibit *Ptbb-6*::*GFP* induction by *isp-1(qm150)* worms. Quantitative fluorescence imaging data is provided in panel F. (n = 4–7 worms per condition; asterisks indicate significantly (*p<0*.*025)* different relative to vector-treated animals).

A number of genes function epistatically to ATFS-1 in response to various forms of mitochondrial disruption and this has been linked to their role in synthesizing mevalonate (*hmgs-1*) and ceramide (*ran-4*, *sptl-1* and F40F12.7) [26]. Both *hmgs-1* and *sptl-1* have also been previously reported to be required for other cellular surveillance responses, including induction of *Pgst-4*::*GFP* upon treatment with azide [60]. To test whether any of these genes are also required for induction of *tbb-6*, we assayed the effect of RNAi knockdown of each on our *isp-1(qm150)* transcriptional reporter lines (Table 1 and S3 Table). As reported, RNAi against *hmgs-1*, *ran-4*, *sptl-1* and F40F12.7 largely blocked *Phsp-6*::*GFP* induction by *isp-1(qm150)*. The effect of these same RNAi on *Ptbb-6*::*GFP* expression was strikingly different. Like loss of *atfs-1*, neither *hmgs-1*, *ran-4* nor *sptl-1* were required for *Ptbb-6*::*GFP* induction (Fig 5A) and knockdown of *hmgs-1* or *sptl-1* further upregulated *Ptbb-6*::*GFP* (quantified in S8 Fig). Only F40F12.7 RNAi completely blocked *Ptbb-6*::*GFP* induction in *isp-1(qm150)* worms (Figs 5A and S8). The protein encoded by F40F12.7 is predicted to act as a transcriptional coactivator and bears significant orthology with CREB-binding proteins and thus from hereon we will refer to it as CBP-3.

Liu and colleagues reported that loss of *Phsp-6*::*GFP* expression in animals treated with *cbp-3* (F40F12.7), *ran-4* or *sptl-1* RNAi could be rescued by exogenous application of C24 ceramide [26]. Using the *cbp-3* RNAi, we replicated this effect on *Phsp-6*::*GFP* expression in *isp-1(qm150)* worms. In contrast, ceramide had no effect on the recovery of *Ptbb-6*::*GFP* expression (Fig 5B). Thus, of all the genes reported to be epistatic to *atfs-1* and the UPR^mt^, only *cbp-3* is also required for *tbb-6* activation, but in a manner independent of ceramide.

### Innate Immune Response Regulators Are Not Required for *tbb-6* Pathway Induction

Many pathogens secrete toxins that interfere with mitochondrial function [26]. Consequently, *C*. *elegans* respond to mitochondrial dysfunction as a pathogen attack and indeed the UPR^mt^ activates genes involved in innate immunity [38, 39]. Numerous other signaling pathways have reported roles in pathogen response. To test whether *tbb-6* might be part of an immune response separate from the UPR^mt^, we assayed *Ptbb-6*::*GFP* expression in *isp-1(qm150)* worms upon RNAi knockdown of four genes reported to mount cellular defenses against infection: *elt-2* [69], *fshr-1* [70], *hsf-1* [71] and *zip-2* [72]. None of these treatments diminished *Ptbb-6*::*GFP* expression (Table 1 and S3 Table), further suggesting that *tbb-6* marks a novel branch of the cell surveillance system.

### Transcription Factors Known to Be Required for Mit Mutant Life-Extension Are Dispensable for *tbb-6* Pathway Induction

Since several transcription factors have already been implicated in the life extension of *isp-1(qm150)* worms [21, 33, 67, 81, 82], we tested whether any are required for *tbb-6* expression. The genes we tested included: *aha-1*, *ceh-18*, *ceh-23*, *cep-1*, *hif-1*, *hlh-30*, *jun-1*, *nhr-27*, *nhr-49*, and *taf-4*. RNAi knock-down of each showed no attenuation of *Ptbb-6*::*GFP* expression in *isp-1(qm150)* worms (Table 1, see also S3 Table). Indeed, some of these RNAi treatments resulted in further upregulation of *Ptbb-6*::*GFP*; most notably, *taf-4* RNAi dramatically upregulated intestinal *Ptbb-6*::*GFP* in *isp-1(qm150)* but not in otherwise wild-type worms.

### Neither Octopamine nor Dopamine Modulates *tbb-6* Expression

Durieux and colleagues demonstrated that mitochondrial disruption confined to neurons was sufficient to both increase lifespan and induce a UPR^mt^ response cell non-autonomously in the intestine [45]. Recently, Burkewitz and colleagues showed that mitochondrial morphology in worms is modulated by neurotransmitters; specifically, when neurons perceive a low energy state via AMPK signaling, neuronal octopamine release is switched off, causing mitochondria in distal tissues to assume a more fused and elongated morphology [83]. Similar mitochondrial morphology has been previously reported in Mit mutants [12]. Taken together, these observations suggest that neuronal mitochondrial dysfunction may alter mitochondrial morphology and lifespan of the whole worm through neurotransmitter or neurohormonal signaling. Furthermore, it is possible that *tbb-6* upregulation in the gut may not be the result of local mitochondrial disruption but of signaling from neurons with compromised mitochondria.

We tested the involvement of octopamine and related neurotransmitters in *tbb-6* regulation via complementary approaches. First, we simply increased neurotransmitter availability in *isp-1(qm150)* worms through exogenous application of octopamine, dopamine, and L-tyramine. Second, we removed these neurotransmitters/neurohormones by crossing our *Ptbb-6*::*GFP* reporter into *cat-2(e1112)* and *tdc-1(ok914)* mutant backgrounds—genes required for synthesis of dopamine and octopamine, respectively—and asked if there was constitutive reporter activation. In short, neither treatment affected *Ptbb-6*::*GFP* expression. Specifically, when *isp-1(qm150); Ptbb-6*::*GFP* reporter worms of various larval stages were transferred to plates supplemented with octopamine, dopamine, or L-tyramine, and GFP expression subsequently monitored over several days, under no condition was *Ptbb-6*::*GFP* expression altered relative to untreated control animals (Fig 5C). Likewise, absence of *tdc-1* or *cat-2* did not constitutively induce *Ptbb-6*::*GFP*, nor did it enhance *Ptbb-6*::*GFP* expression in animals fed *isp-1* RNAi relative to control-treated worms (Fig 5D). Finally, *unc-13* is required for neurotransmitter release [79]. When we treated *isp-1(qm150); Ptbb-6*::*GFP* worms with *unc-13* RNAi, we again observed no diminution of *Ptbb-6*::*GFP* reporter expression (Table 1). We conclude that neither octopamine, dopamine nor L-tyramine modulates *tbb-6* expression.

### Inhibition of the Mitochondrial-Associated Degradation (MAD) Pathway Blocks TBB-6::GFP Induction

Segref and colleagues [84] have presented evidence for a novel cell surveillance mechanism that is active in both human cells and worms following mitochondrial respiratory dysfunction. They showed that activity of the ubiquitin/proteosome system (UPS) is specifically repressed in the cytosol following insult to various mitochondrial respiratory chain and matrix bioenergetic targets, and that this response is strongly exacerbated by removal of SKN-1. This reduction in cytosolic UPS activity was not due simply to exhaustion of ATP levels, instead UPS activity could be recovered by increasing the assembly and activity of the 26S proteosome, or by addition of N-acetyl cysteine. These findings indicate that the 26S proteosome becomes limiting under conditions of mitochondrial bioenergetic stress, and the authors speculated that the 26S proteosome was re-directed to the outer mitochondrial membrane (OMM) as part of the Mitochondrial-associated Degradation (MAD) pathway. The MAD pathway functions analogously to the endoplasmic reticulum-associated degradation (ERAD) pathway [85] to retrieve and degrade dysfunctional OMM proteins [86, 87]. Both pathways utilize overlapping machinery, in particular the conserved AAA-ATPase Ccd48/VCP/p97, as well as the ubiquitin-binding and Ccd48-binding heterodimeric cofactor UFD1/NPL4, to dislodge ubiquitinated proteins from each respective organelle and chauffeur them to the 26S proteosome for degradation. Specificity is obtained through additional co-factors that recognize ubiquitinated proteins in each compartment and also bind to the core complex. Wu and colleagues [74], recently confirmed using yeast that disruption to the mitochondrial respiratory chain, the matrix protein folding environment, or mitochondrial oxidative stress, are all sufficient to strongly activate the MAD pathway.

We tested if MAD pathway activity plays a role in controlling the expression of TBB-6::GFP in *isp-1(qm150)* worms. *ufd-1* encodes the sole UFD1 ortholog in *C*. *elegans*. Yeast two-hybrid analyses have shown this protein interacts with both CDC-48.1 and CDC-48.2, as well as NPL-4.2 [88]. RNAi-mediated inhibition of all four genes reduced *Ptbb-6*::*GFP* expression (Fig 5E and 5F and Table 1). These findings imply that the signal for *Ptbb-6*::*GFP* reporter activation is downstream of MAD pathway activation and they raise the intriguing possibility that reduced UPS activity in the cytosol might result in stabilization and activation of a cytosolic signaling pathway that ultimately leads to upregulation of *tbb-6* expression.

### *tbb-6* Is Under MAPK Control

Mitogen activated protein kinase (MAPK) cascades are conserved across eukaryotes as cytosolic signaling pathways that respond both to mitogens and to stressful stimuli. The p38 family of MAPKs respond to a variety of stressors and play an integral role in activating the immune response [89]. *C*. *elegans* is no exception; the p38 MAPK, PMK-1, is crucial to immunity [90] and activates both SKN-1 (Nrf2) [91] and DAF-16 (FOXO) [92] in response to oxidative stress. The other family of stress-activated protein kinases are the c-Jun N-terminal kinases (JNKs), which perform a vast repertoire of functions [93] and may mediate the mammalian UPR^mt^ [42]. One of the *C*. *elegans* JNKs, KGB-1, is involved in cellular surveillance and pathogen aversion [38], and acts in a competitive manner with the UPR^mt^ [41].

**Fig 6:**
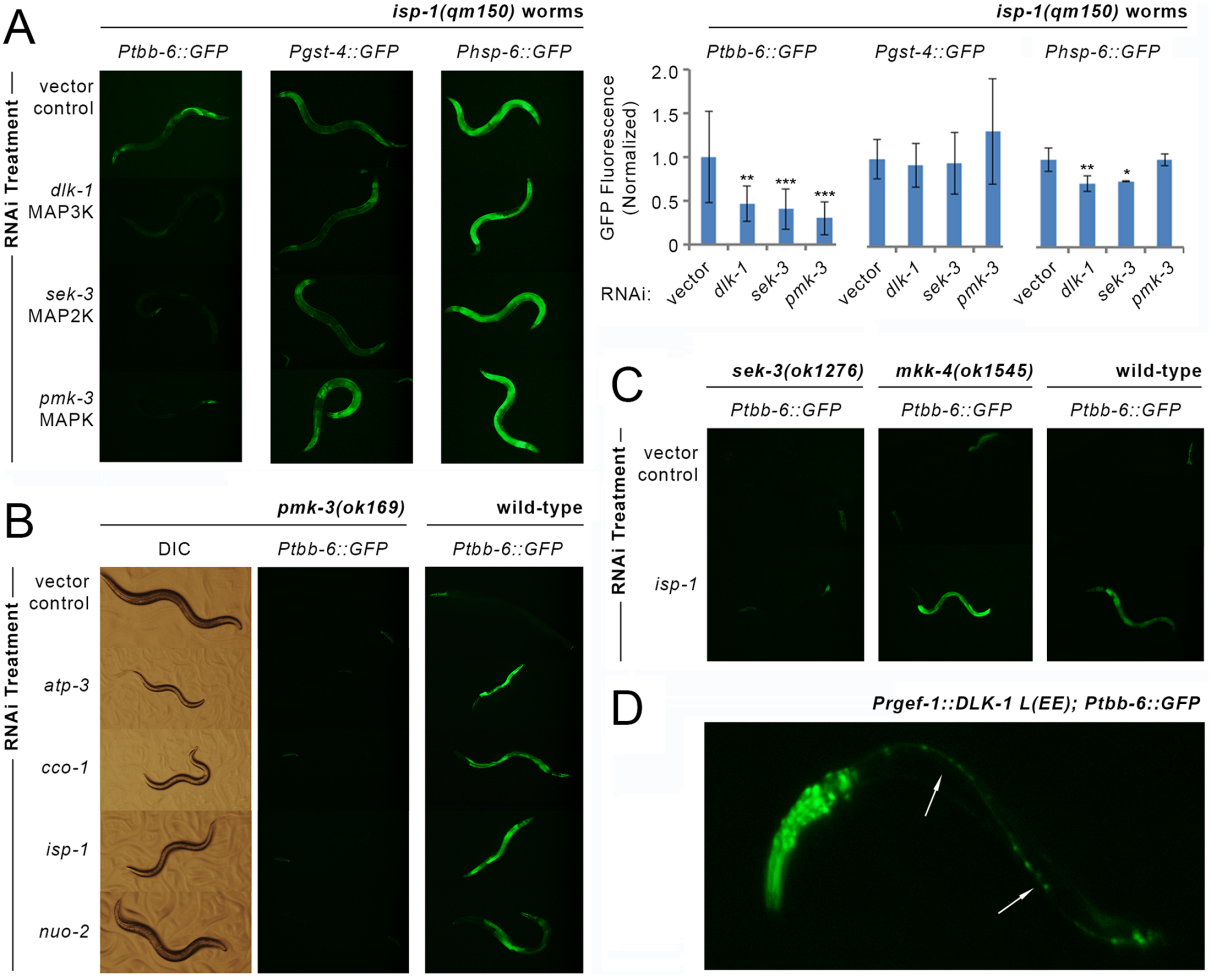
*Ptbb-6*::*GFP* expression requires a MAPK signal cascade. (**A**) RNAi-mediated disruption of the MAP3K/MAP2K/MAPK pathway defined by DLK-1 → SEK-3 → PMK-3 blocks induction of *Ptbb-6*::*GFP* in *isp-1(qm150)* worms but not *Pgst-4*::*GFP* nor *Phsp-6*::*GFP* reporter expression. Graph provides quantification of reporter expression level, normalized to vector-control RNAi (Mean+/-SD, *n =* 12–18 worms/ RNAi treatment). Asterisks indicate significant difference relative to vector (one-way ANOVA and *ad hoc* using Dunnett’s Multiple Comparisons Test, *p<0.05, **p<0.01, ***p<0.001). See also S9 Fig and S3 Table. (**B**) *pmk-3(ok169)* null mutants show the expected reduction in size upon RNAi knockdown of mitochondrial respiratory subunits, but are incapable of inducing *Ptbb-6*::*GFP*. (**C**) MKK-4 is not required for *Ptbb-6*::*GFP* induction following mitochondrial ETC disruption by *isp-1* RNAi, unlike SEK-3. (**D**) Neuron-specific expression of a constitutively active form of DLK-1 acts cell autonomously to activate *Ptbb-6*::*GFP* expression. There is no induction of *Ptbb-6*::*GFP* in intestinal or other cells. (Arrows mark ventral nerve cord).

We tested for a role of MAPK signaling in *tbb-6* expression by assaying whether RNAi-mediated knockdown of each of the 14 known *C*. *elegans* MAPKs blocked reporter induction in *isp-1(qm150); Ptbb-6*::*GFP* worms (Table 1). Significantly, *pmk-3* RNAi alone completely blocked *Ptbb-6*::*GFP* expression (Fig 6A). Knockdown of two uncharacterized MAPKs had a weak inhibitory effect (C05D10.2, F09C12.2), while *jnk-1* and *sma-5* further upregulated *Ptbb-6*::*GFP* expression. All other MAPKs were without effect. The requirement for *pmk-3* in *Ptbb-6*::*GFP* activation was not unique to *isp-1(qm150)* mutants; it was also readily apparent in *isp-1(qm150); ctb-1(qm189)* worms (**Fig A in** S9 Fig), and even in *nuo-6(qm200)* worms which only show weak *Ptbb-6*::*GFP* induction (**Fig B in** S9 Fig). Moreover, using a reciprocal approach, *pmk-3(ok169)* mutants containing the *Ptbb-6*::*GFP* reporter failed to induce GFP when cultured on various RNAi targeting subunits of the mitochondrial ETC, including *isp-1* (Fig 6B and **Fig C in** S9 Fig). Notably, while inactivation of *pmk-3* completely blocked *Ptbb-6*::*GFP* induction outside the pharynx, it had no effect on *Phsp-6*::*GFP* expression in either *isp-1(qm150)* (Fig 6A) or *nuo-6(qm200)* mutant animals (**Fig B in** S9 Fig), and it further upregulated *Pgst-4*::*GFP* in *isp-1(qm150)* worms that previously only showed moderate induction of this reporter (Fig 6A). We next used RNAi to map additional upstream elements of the *pmk-3* MAPK signaling cascade and tested all 10 known MAPK kinases (MAP2K), and 12 MAPK kinase kinases (MAP3K) (Table 1). We found the uncharacterized MAP2K *sek-3*, and the well characterized MAP3K *dlk-1*, both to be unequivocally required for *Ptbb-6*::*GFP* upregulation in *isp-1(qm150)*, *isp-1(qm150); ctb-1(qm189)* and *nuo-6(qm200)* animals (Figs 6A, S9A and S9B). Knockdown of four other MAP2Ks had milder effects on *Ptbb-6*::*GFP* induction: knockdown of *mkk-4* consistently increased reporter expression while the expression phenotype produced by *mek-2* knockdown was highly variable, with some worms very dark and others very bright. Knockdown of either *jkk-1* or *sek-1* both reduced *Ptbb-6*::*GFP* expression, but not to the extent produced by *sek-3* knockdown. (Table 1). Thus, we conclude that a novel MAPK cascade consisting of DLK-1, SEK-3 and PMK-3 is required in worms for mitochondrial bioenergetic disruption to induce *Ptbb-6*::*GFP*.

Both DLK-1 and PMK-3 play important roles in axon and synapse development [65, 94, 95] as well as efficient axon regeneration [96]. In these capacities, DLK-1 and PMK-3 function with the MAP2K, MKK-4 [65]. While our RNAi-based approach for identifying MAP2Ks essential for *Ptbb-6*::*GFP* expression in *isp-1(qm150)* mutants did not detect a role for *mkk-4*, but instead *sek-3* (Table 1), we independently verified this result using *mkk-4(ok1545)* and *sek-3(ok1276)* loss-of-function mutants. We crossed our *Ptbb-6*::*GFP* reporter into both mutant backgrounds and monitored GFP induction when worms were treated with *isp-1* RNAi. Consistent with our earlier observation, only loss of *sek-3*, and not *mkk-4*, abrogated GFP expression. The *mkk-4(ok1545)* mutation, in fact, enhanced *Ptbb-6*::*GFP* reporter expression over and above that of control worms (Fig 6C).

It has been reported previously that a DLK-1::GFP translational fusion reporter, expressed under the control of the endogenous DLK-1 promoter, localizes specifically to neurons, accumulates in axonal boutons, and is tightly controlled by the E3 ubiquitin ligase, *rpm-1* [62]. We have shown that expression of our *Ptbb-6*::*GFP* transcriptional reporter in *isp-1(qm150)* mutants is strongly activated in intestinal cells, and less so in neurons (Fig 2). This expression occurs in a *dlk-1*, *sek-3* and *pmk-3* dependent manner (Fig 6A–6C). To determine whether neuronal DLK-1 signaling functions non-cell autonomously to mediate the intestinal expression of *Ptbb-6*::*GFP*, we expressed a constitutively-active form of DLK-1 [62] exclusively in the neurons of *Ptbb-6*::*GFP* reporter worms. Under these conditions, *Ptbb-6*::*GFP* fluorescence was detected only in neuronal cells and not in the intestine (Fig 6D), opening the intriguing possibility that DLK-1 is expressed in cells outside of neurons or is activated differently under conditions of mitochondrial dysfunction (see Discussion).

### Hyperactivation of PMK-3 Arrests Mit Mutant Development

While Mit mutants typically exhibit a collection of co-segregating phenotypes—including delayed development, smaller size, and extended lifespan, these phenotypes can, in fact, be separated [20]. This was first demonstrated by the discovery of the *isp-1(qm150); ctb-1(qm189)* double mutant which, as previously mentioned, exhibits an attenuated delay in development but the same extended lifespan as *isp-1(qm150)* [54]. We proceeded to test for a role of PMK-3 in development and lifespan.

**Fig 7:**
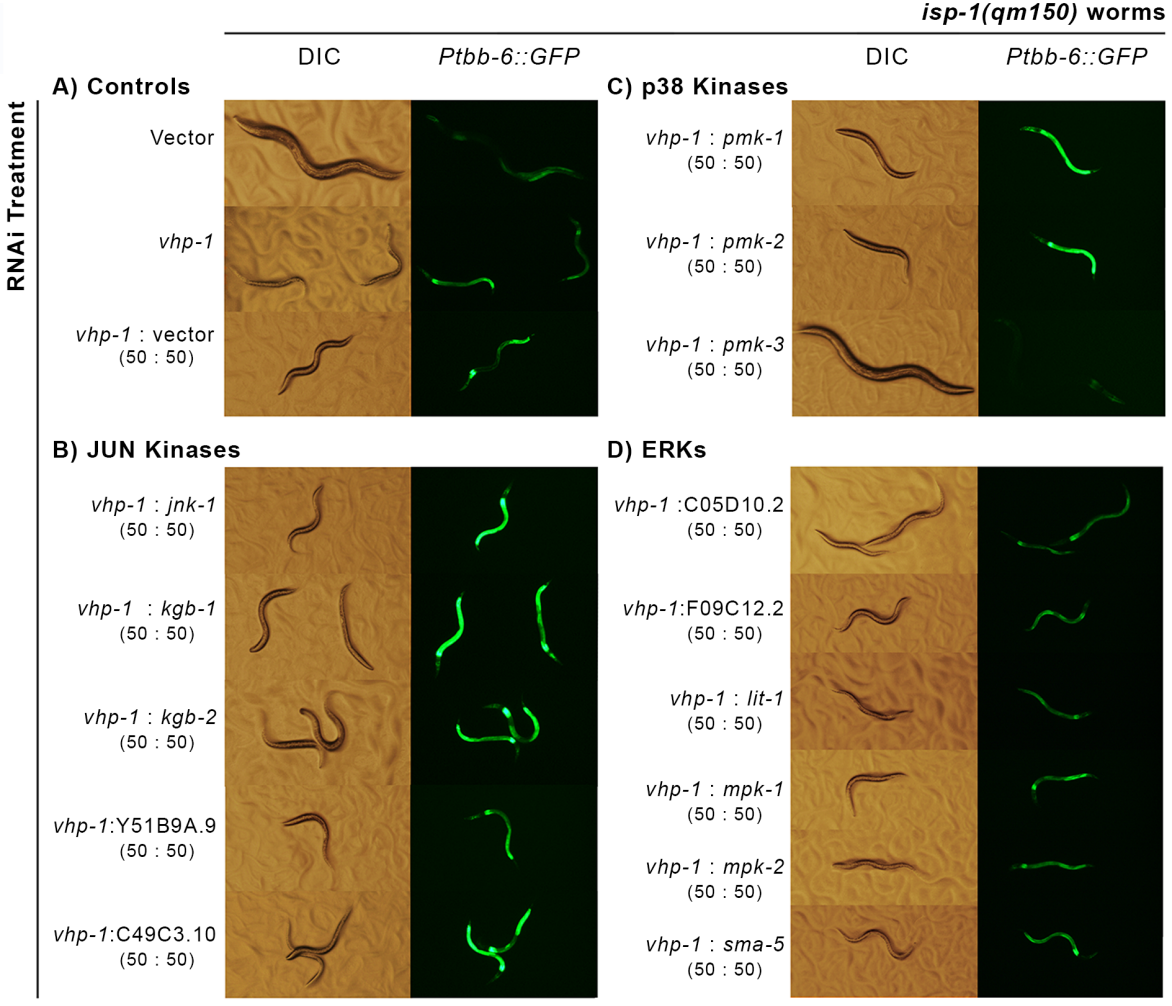
A single MAPK is required for the induction of *Ptbb-6*::*GFP* following mitochondrial bioenergetic disruption. (**A)** RNAi knockdown of the dual-specificity phosphatase *vhp-1* in *isp-1(qm150)* worms leads to hyperactivation of *Ptbb-6*::*GFP* and L3 larval arrest. (**B-D)** Inhibition of *pmk-3* uniquely rescues both larval arrest and blocks reporter expression among all fourteen JNK (B), p38 (C) and ERK-type (D) MAPKs.

RNAi knockdown of *pmk-3* neither accelerated nor delayed the development of either *isp-1(qm150)* or *nuo-6(qm200)* Mit mutants. To test the effect of further upregulation of PMK-3, we reasoned that knocking down a negative regulator of MAPKs should result in hyperactivation of PMK-3. Dual-specificity phosphatases (DUSPs) act as negative regulators of MAPKs [97]. We used BLAST to identify potential DUSPs in *C*. *elegans* and assayed *isp-1(qm150)* development and *Ptbb-6*::*GFP* induction upon RNAi knockdown of each (Table 1). Most treatments had no effect on either phenotype. Of the two that did, the most dramatic was knockdown of VHP-1, a DUSP known to act preferentially on the stress-activated protein kinases—the JNKs and p38s. Significantly, *vhp-1* RNAi dramatically further upregulated *Ptbb-6*::*GFP* and arrested both *isp-1(qm150)* and *nuo-6(qm200)* worms at the L3 larval stage (Figs 7 and S10A). Upregulation of *Ptbb-6*::*GFP* by *vhp-1* RNAi was also observed in *isp-1(qm150); ctb-1(qm189)* mutants (**Fig A in** S9 Fig). This response was specific to the context of mitochondrial disruption, as wild-type worms cultured on *vhp-1* RNAi displayed only minimal hypodermal induction of *Ptbb-6*::*GFP* (S11 Fig) and, as has been previously reported, did not arrest but matured into smaller adults [98]. To confirm that the arrest of *isp-1(qm150)* worms upon *vhp-1* knockdown was due to hyperactivation of PMK-3, we assayed both *isp-1(qm150)* and wild-type worm development following simultaneous knockdown of *vhp-1* in combination with either of the 14 annotated worm MAPKs (Figs 7 and S11). No MAPK RNAi had any effect on *vhp-1* arrest with the notable exception of *pmk-3*, which resulted in a near total rescue of the phenotype; that is, *isp-1(qm150)* worms grown on a 1:1 combination of *vhp-1* and *pmk-3* RNAi by-passed L3 larval arrest and matured into fertile adults (Fig 7). It is possible that use of a combination RNAi approach differentially reduced the efficacy of *vhp-1* RNAi specifically in combination with *pmk-3* RNAi; this too would permit worms to continue development. To exclude this possibility, we constructed a *nuo-6(qm200); pmk-3(ok169)* double mutant and then examined its ability to proceed through development when cultured on full strength *vhp-1* RNAi. Like *isp-1(qm150)* mutants, *nuo-6(qm200)* single mutants normally arrest under these conditions. Genetic removal of *pmk-3*, however, allowed these worms to by-pass larval arrest and produce offspring (**Fig A in** S10 Fig).

### PMK-3 Is Required for Mit Mutant Life Extension

**Fig 8:**
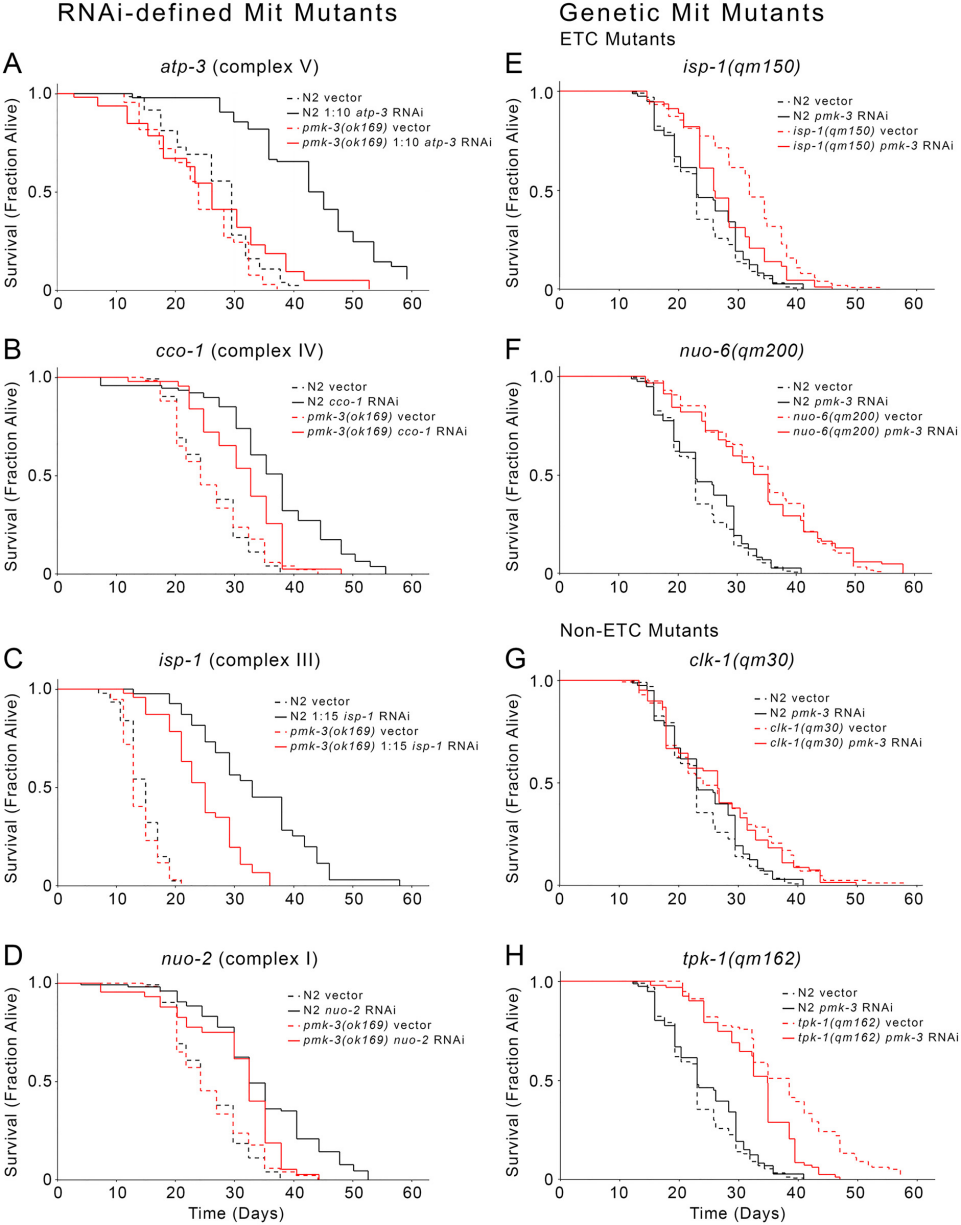
Role of *pmk-3* in Mit mutant life extension. (**A-D**) *pmk-3(ok169)* worms showed significantly attenuated life extension upon RNAi knockdown of specific mitochondrial ETC subunits. (**E-H**) *isp-1(qm150)* and *tpk-1(qm162)*, but not *nuo-6(qm200)* or *clk-1(qm30)* mitochondrial ETC mutants display significantly attenuated life extension following RNAi-mediated removal of *pmk-3*. For these four panels, lifespan curves represent averages of two or more independent experiments. Significance values, N, and lifespan statistics are provided in S4 Table. Raw lifespan data is provided in S5 Table.

We next tested whether PMK-3 is required for Mit mutant longevity, again using independent approaches through use of both genetic and reciprocal RNAi-mediated mitochondrial disruption. We first treated wild-type and *pmk-3(ok169)* null worms with RNAi targeting *nuo-2* (complex I), *isp-1* (complex III), *cco-1* (complex IV), or *atp-3* (complex V). Life extension of wild type worms on these particular RNAi constructs has been well-characterized by us and others [13, 20, 99]. By itself, the *pmk-3(ok169)* null mutation had no effect on lifespan, yet on each of the four RNAi treatments, *pmk-3* null worms had significantly attenuated life extension relative to wild-type animals (Fig 8A–8D). The effect of the *pmk-3* mutation on *atp-3* RNAi was especially pronounced, with one replicate showing a complete absence of life extension (Fig 8A and S4 Table).

**Fig 9:**
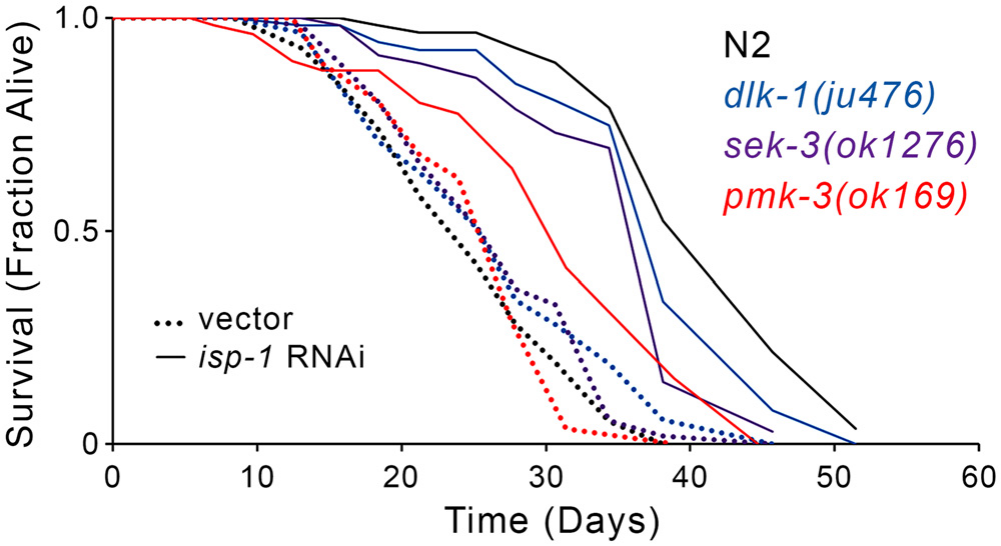
*dlk-1*, *sek-3* and *pmk-3* are required for life extension following RNAi-mediated disruption of *isp-1*. Mutations in *dlk-1(ju476)*, *sek-3(ok1276)* and *pmk-3(ok169)* attenuate the life extending effects of *isp-1* RNAi relative to wild type (N2) worms (N = 60 worms/condition). Full lifespan statistics in S4 Table.

We next used a reciprocal approach to test the effect of *pmk-3* RNAi on the lifespan of four genetically-defined Mit mutants: *isp-1(qm150)*, *nuo-6(qm200)*, *clk-1(qm30)* and *tpk-1(qm162)*. The latter two mutants indirectly affect the mitochondrial electron transport chain [100]: *clk-1* encodes demethoxyubiquinone mono-oxygenase, an enzyme required for ubiquinone biosynthesis, while *tpk-1* disrupts the TCA cycle by limiting thiamine, which is essential for α-ketoacid dehydrogenase activity. We found that *pmk-3* knockdown significantly (*p-value* < 0.001) attenuated the life extension of *isp-1(qm150)* and *tpk-1(qm162)* mutants, but had no effect on either *clk-1(qm30)* or *nuo-6(qm200)* animals (Fig 8E–8H). Intriguingly, the selective requirement for *pmk-3* in the life extension of only some Mit mutants correlated both with the specific respiratory complex that was targeted, and induction of *Ptbb-6*::*GFP*. For example, *pmk-3* knockdown had moderate to no effect on the lifespan of animals with disrupted complex I activity, namely *nuo-6(qm200)* mutants and worms with RNAi-induced *nuo-2* knockdown, which only moderately or weakly induce *Ptbb-6*::*GFP*, respectively, in line with the effect on lifespan following *pmk-3* removal. In contrast, *pmk-3* knockdown significantly attenuated life extension in the context of complex III, IV or V disruption, as in *isp-1(qm150)* mutants and worms with RNAi knockdown of *isp-1*, *cco-1* or *atp-3*, all conditions which strongly induce *Ptbb-6*::*GFP*. Finally, we tested the effect of knocking out the upstream components of the MAPK cascade on lifespan following complex III disruption. Similar to *pmk-3(ok169)*, both *dlk-1(ju476)* and *sek-3(ok1276)* mutants have lifespans close to wild-type animals cultured on vector alone, but have dramatically attenuated life extension when exposed to *isp-1* RNAi (Fig 9), emphasizing the specific requirement of this pathway for longevity in the face of mitochondrial disruption. Significantly, while several genes have been found to be required for Mit mutant longevity (Table 1), this is the first demonstration of a mitochondrial stress response required for life extension in relation to specific forms of mitochondrial dysfunction.

### TBB-6 Plays a Minor Role in Life Extension following PMK-3 Activation

**Fig 10:**
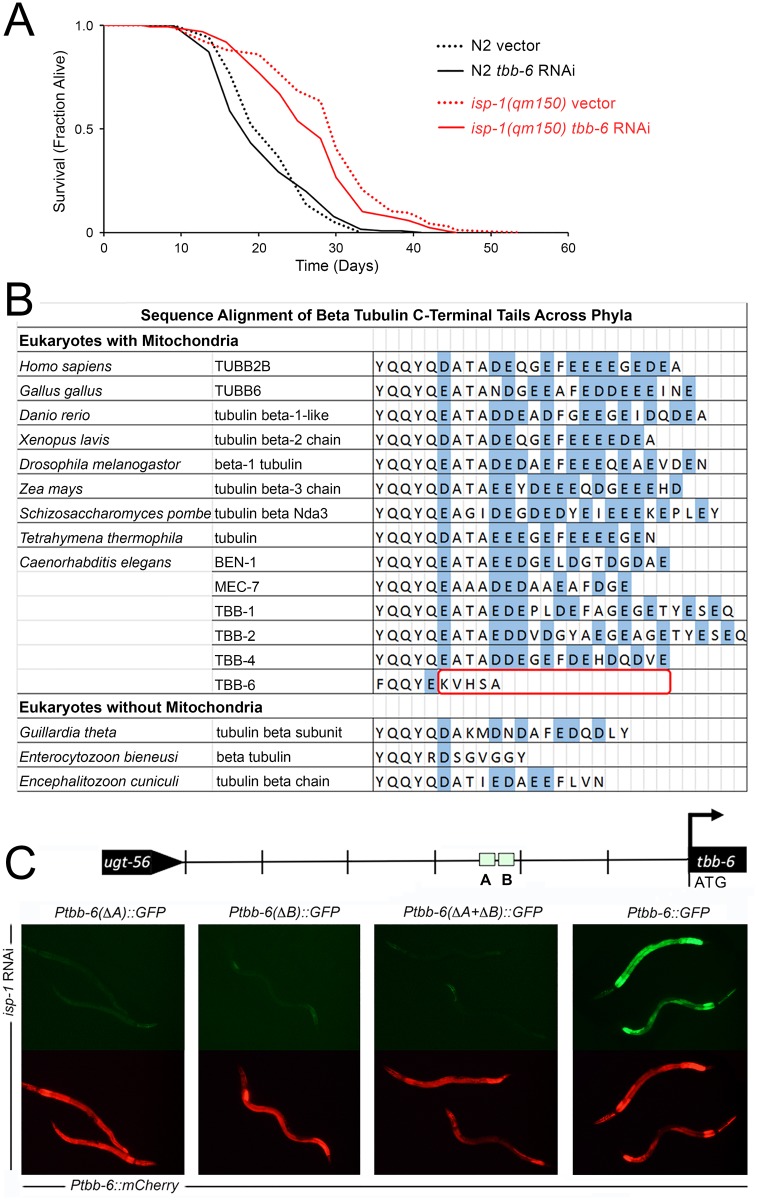
RNAi-mediated inhibition of *tbb-6* mildly inhibits the life extension of *isp-1(qm150)* Mit mutants—potentially by mediating voltage-dependent anion channel (VDAC) activity. (**A)** Survival analysis of *isp-1(qm150)* and wild type (N2) worms cultured on RNAi to *tbb-6* or vector control (pL4440). The lifespan of *isp-1(qm150)* mutants is reduced by ~7% following knockdown of *tbb-6*. (Combined data from replicate experiments, Log rank test *p* < 0.003, N = 126–206 worms/condition). Full lifespan statistics in S4 Table. (**B**) C-termini of β-tubulins from various species. Non-*C*. *elegans* data derived from Fig 6A of Rostovtseva and colleagues [101]). (**C**) Removal of either of the two C/EBP-like motifs in the *tbb-6* promoter of *Ptbb-6*::*GFP* abrogates GFP reporter expression. Transgenic lines contain mCherry under the control of the wild-type *tbb-6* promoter as an internal control.

We have used *Ptbb-6*::*GFP* throughout this study as a marker of activation of a potential mitochondrial retrograde response. Expression of this reporter positively correlates with life extension across multiple ETC disruptants (compare S1 Fig and Fig 8), and *tbb-6* was one of the most highly upregulated of all genes following *spg-7* RNAi treatment (Fig 1A). We tested whether *tbb-6* itself plays a role in life extension following ETC disruption. When *isp-1(qm150)* worms were cultured on *tbb-6* RNAi, a mild (~7%) but significant (*p < 0*.*01*) reduction in lifespan was observed (Fig 10A). We speculate that TBB-6 may have a function in regulating ADP entry into mitochondria (Fig 10B, and Discussion).

### A Role for CCAAT/Enhancer Binding Proteins in PMK-3 Signaling

We identified two C/EBP-like motifs present in the promoter of *tbb-6* (Fig 1E). As a first step toward identifying transcription factors that function downstream of PMK-3, we tested whether either of these motifs was required for *Ptbb-6*::*GFP* activation following mitochondrial ETC disruption. We used site-directed mutagenesis to selectively remove each site, as well as both sites together. These mutated promoters were then coupled to GFP, and finally co-injected into worms along with mCherry expressed under control of the wild-type *tbb-6* promoter. As expected, removal of both promoter elements completely abolished the ability of *isp-1* RNAi to induce GFP (Fig 10C).

## Discussion

In this study we have identified a novel MAPK cascade which is required in worms for life extension following mitochondrial bioenergetic dysfunction. We do not know whether this signaling cascade simply acts during development and is essentially a permissive factor that allows mitochondrial retrograde response signaling to occur, whether the cascade functions as a *bona fide* retrograde response that controls longevity directly, or whether it forms part of a signaling pathway that is activated in distal cells as a consequence of mitochondrial dysfunction in unrelated tissues (that is, cell non-autonomous signaling). At present we favor the notion that DLK-1, SEK-3 and PMK-3 function as a true retrograde response based on the following supporting evidence: (i) *tbb-6* is the most highly upregulated gene among the 148 *atfs-1* independent gene set that we initially described. When we coupled GFP to a copy of the *tbb-6* promoter and treated worms with various ETC insults, this reporter was most strongly expressed in the gut, the same tissue that the bona fide UPR^mt^ reporter *Phsp-6*::*GFP* was activated, suggesting *tbb-6* is activated in cells directly experiencing mitochondrial stress (Fig 2). (ii) The novel DLK-1, SEK-3 and PMK-3 stress cascade, which we show is essential for *tbb-6* induction, functions cell autonomously; that is, when we constitutively activated DLK-1 in neurons we observed expression of *Ptbb-6*::*GFP* only in neurons (Fig 6D). (iii) The Mitochondrial-associated degradation (MAD) pathway functions within cells experiencing mitochondrial dysfunction to extract and remove damaged outer mitochondrial membrane proteins. Inhibiting core elements of this pathway should exacerbate mitochondrial dysfunction and enhance any stress signaling to distal tissues. Despite this, RNAi-mediated inhibition of MAD components in *isp-1(qm150)* mutants did not enhance *Ptbb-6*::*GFP* expression, rather, it suppressed it (Table 1). This finding argues that *tbb-6* is induced cell autonomously following MAD pathway activation in cells directly experiencing mitochondrial damage.

Promoter analysis of the 148 *atfs-1* independent genes identified in this study revealed significant enrichment of two transcription factor binding sites in essentially non-overlapping gene sets (Fig 1). One group of 42 genes shared a DNA motif closely related to the DNA binding site of mammalian CCAAT/enhancer binding proteins (C/EBP transcription factors). We showed that this motif is used in signaling mitochondrial ETC stress as its removal from the promoter of the *Ptbb-6*::*GFP* reporter blocked induction by *isp-1* RNAi. A second group of 40 genes contained an EOR-1 binding element. Future studies will address the role of EOR-1 in Mit mutant longevity, which is a likely proposition given that EOR-1 is an essential component of a recently-described longevity response mediated by EGF in adult worms [102, 103]. Suffice to say, we have found that RNAi to EOR-1 does not block *Ptbb-6*::*GFP* expression, raising the possibility that more than one signaling pathways may function in the longevity control of Mit mutants. In this regard, the genes under EOR-1 control that are essential for the longevity response mediated by EGF in adults worms [103], are over-represented in our 148 gene set (10 out of a total of 503 up- or down-regulated genes, hypergeometric probability <0.003). Recently, EOR-1 was also implicated in the genetic response to dietary restriction and the set of genes under its control were enriched in mitochondrial targets [104]. Moreover, while DAF-16 binding elements were not significantly enriched in our 148 gene set, we nonetheless found 27 genes that contained DAF-16 binding sites (Fig 1D). Kumar and colleagues [51] recently described a signature set of 37 genes that directly bound DAF-16 in all DAF-16 chromatin-binding studies to date. It has been repeatedly shown that DAF-16 is not required for the Mit phenotype yet, surprisingly, four of these 37 core DAF-16 binding genes are present in our set of 148 *atfs-1* independent genes. Based on our sample size, this is unlikely to have occurred by chance (hypergeometric probability <0.0002). We predict that EOR-1 and the transcription factor(s) that binds the CCAAT/enhancer binding protein site, will work in concert to turn on a novel kind of hybrid stress response in Mit mutants. If true, this idea would be in line with the remarkable study of Stroustrup and colleagues [105] that showed Mit mutants in particular (and to a lesser extent, dietary restriction), did not simply temporally scale lifespan, as various other genetic and environmental interventions that also extend life did, but instead fundamentally changed the way worms age. In further support of such a possibility, a search for enriched functional GO terms among the 148 *atfs-1* independent genes using DAVID [106], revealed a significant (q-value < 0.05) enrichment of genes encoding FBOX-containing proteins (8 genes), small heat shock proteins (sHSPs, 6 genes), and gene clusters involved in aging (12 genes), ER-nuclear signaling and cytochrome P450 activity. FBOX proteins are components of SCF ubiquitin E3 ligase complexes that play important roles in protein turnover [107], along with sHSPs. The FBOX cluster was enriched in genes containing the CCAAT motif, while the sHSPs and other clusters were enriched in genes containing EOR-1 motifs.

Based on the well-established roles of other MAPKs, we speculate that PMK-3 controls the activity of one or more transcription factors. Again, we do not know if DLK-1, SEK-3 and PMK-3 function prior to, or after, mitochondrial bioenergetic stress, but if it is after then we predict likely targets could be members of the CCAAT/enhancer binding proteins (C/EBP transcription factors), since removal of either of the two C/EBP-like binding motifs in the promoter of *tbb-6* blocked *Ptbb-6*::*GFP* reporter induction, which we also showed is dependent upon PMK-3. In *C*. *elegans*, there are three transcription factors orthologous to mammalian C/EBPs, namely, CEBP-1, CEBP-2, and ZIP-4. These belong to a broader category of transcription factors known as bZIPs for the basic leucine zipper domain which binds the DNA. Intriguingly, both ATFS-1 and SKN-1 are themselves bZIP transcription factors, suggesting possible mechanisms for the complementary nature with our novel retrograde pathway: ATFS-1 may bind and compete with C/EBP-like proteins for the same promoter elements, or they might share a common protein binding partner. We have already tested the bZIP *jun-1* for its known requirement in Mit mutant longevity [33] and *cebp-1* for its known role with *pmk-3* in neuron morphology [108]. Since neither of these diminished *Ptbb-6*::*GFP* activation by *isp-1(qm150)*, our efforts will now be focused on the remaining bZIP transcription factors in *C*. *elegans*.

In mammals, C/EBPδ is known to act in a calcium-activated, mitochondrial retrograde response [109], raising the possibility that increases in cytoplasmic calcium following mitochondrial depolarization could also be involved in the novel MAPK pathway that we have identified in this study. Interestingly, C/EBP proteins are known to recruit CREB-binding protein (CBP) [110]. One possible function for CBP-3, the CBP ortholog which we found to be essential for *Ptbb-6*::*GFP* induction in this study, might be that it is needed to directly interact with transcription factors that bind to C/EBP motifs. If CBP-3 functions downstream of PMK-3, one prediction is its removal by RNAi should permit *isp-1* animals cultured on *vhp-1* RNAi to resume larval development, analogous to co-treatment with *pmk-3* RNAi. We have found this not to be the case, although it did block *Ptbb-6*::*GFP* hyperactivation (**Fig B in** S10 Fig). It is difficult to interpret the significance of this result, however, since *cbp-3* RNAi itself causes *isp-1(qm150)* worms to arrest [26] and we found it to result in sterility and early mortality even in wild-type worms. Clearly, *cbp-3* has essential roles, including in ceramide biosynthesis [26], and whether it plays a specific or general role in signaling mitochondrial dysfunction shall require further investigation.

The previously described roles for PMK-3 relate to a MAPK cascade required for both neuronal development [65] and axon regeneration [111]. Interestingly, while this MAPK cascade is also initiated by DLK-1 (MAP3K), it utilizes the MAP2K MKK-4, instead of SEK-3 which we found to be required for *Ptbb-6*::*GFP* induction. Whether these differences in MAP2K usage are mediated by different DLK-1 isoforms or reflect different tissues of activation remains a question for future study. Intriguingly, it was recently shown that sensory neurons of Mit mutants have reduced functionality relative to wild-type animals [112], suggesting there could be competition for DLK-1 by the two MAP2Ks in the same tissue and that increased neuronal response time may be the payoff for long term survival under stress. In our studies, both genetic and RNAi-mediated removal of *mkk-4* failed to reduce *Ptbb-6*::*GFP* expression. The same RNAi construct was employed previously and shown to block SKN-1 activation induced by oxidative stress [113]. These findings further highlight the complementarity between the PMK-3 and SKN-1 signaling pathways that we have discovered in this work. DLK-1 also been implicated in Wallerian degeneration in mammals and flies [114], the active process by which severed axons self-destruct. This is especially interesting because DLK-1 is coupled to JNK activation in this pathway, via MKK4/7 and the NAD+ sensor and adaptor protein SARM1 [115]. Presumably other adaptor proteins could act to modulate DLK-1 target proteins in a different setting, and this may be what is behind the novel DLK-1, SEK-3, PMK-3 signaling pathway that we have identified in this study [116].

MAPK signaling is highly conserved across phyla, and p38 signaling has been implicated in numerous pathologies. However, most studies have looked at the role of the p38α isoform, to the extent that it is referred to simply as p38 in much of the literature [117]. However, the four mammalian p38 isoforms differ in expression across tissues as well as in their substrate specificity, and inhibition of different isoforms can produce opposite effects [118], limiting the potential for broad spectrum p38 inhibitors in ameliorating disease. The complexity of p38 MAPK signaling is similar in worms: The three isoforms exhibit differential tissue specificity and methods of activation [119]. In worms, the p38 that behaves most like the well-studied mammalian p38α is PMK-1, which, as stated previously, is activated by oxidative stress and plays an essential role in immunity. Further study of the other two p38 isoforms in *C*. *elegans* is likely to shed light on the roles of the less studied mammalian isoforms as well.

Finally, while we used *Ptbb-6*::*GFP* as a marker of PMK-3 activity that somehow permitted a functional Mit mutant longevity response following complex III, IV and V disruption, we also showed that *tbb-6* itself is required for life extension following mitochondrial disruption. TBB-6 is unusual among β-tubulins in that its C-terminus is notably truncated relative to other β-tubulins (Fig 9B). Rostovtseva and colleagues [101] have reported that the C-termini of β-tubulins which are enriched in glutamate can plug the voltage-dependent anion channel (VDAC) and reduce ADP entry into mitochondria [101]. This finding raises the intriguing possibility that TBB-6 may function to enhance ADP entry into mitochondria under stressed conditions. The identification of the precise mechanism by which TBB-6 functions to extend life, along with the mode of action of DLK-1, SEK-3 and PMK-3 in mitochondrial stress-induced longevity, stands to be an exciting area for future investigation.

## Methods

### Identification of *atfs-1* Independent Gene Set and Promoter Analysis

Gene Expression Omnibus dataset GSE38196, first described in [43], was used to identify genes upregulated independently of *atfs-1* following mitochondrial dysfunction (*spg-7* RNAi). Full details of our procedure to isolate *atfs-1* independent genes from this dataset is provided in S1 Text. The MEME Suite of tools (v4.10.1) [120] was used to identify enriched DNA elements (ungapped) among the promoter regions of the identified gene subset. We limited our search to 400 bp of the most proximal 5’ sequence of each gene. MAST [121], was used to locate DAF-16 binding sites using a weighted matrix based on the consensus identified by Kumar and colleagues [51].

### Nematode Strains and Maintenance

A complete list of *C*. *elegans* strains used in this study is provided in S6 Table. All strains were maintained at 20°C on standard NGM-agar plates [20].

### Transgene Construction and Transgenic Strain Generation

Recombinant array construction, microinjection procedures and choice of strain background are detailed in S1 Text and S6 Table.

### Feeding RNAi

Feeding RNAi and RNAi dilution studies were performed as previously described [20]. Details regarding either the source or construction of feeding RNAi constructs is provided in Supplemental Experimental Procedures (S1 Text).

### Fluorescence Imaging and Quantification

Images of first day adult worms were captured using an Olympus DP71 CCD camera connected to an Olympus SZX16 fluorescence dissecting microscope. Where relevant, images were quantified using ImageJ software (NIH). A one-way ANOVA, or Student’s t-test with correction applied for multiple testing was employed, as indicated in figure legends.

### *atfs-1* mRNA Quantification

qRT-PCR was used to measure the efficacy of *atfs-1* RNAi knockdown in Fig 4C. Details of strain culturing, mRNA extraction, cDNA synthesis, primer design for qRT-PCR analysis, data normalization and statistical testing are provided in S1 Text.

### Lifespan Analyses

Lifespan studies were performed as described previously [20]. Use of FudR was avoided. The first day of adulthood was designated as day one. Data was analyzed using the log rank test and Cox’s proportional hazard model. A full description of all lifespan experiments is provided in S4 Table. Raw lifespan data is provided in S5 Table.

## Supporting Information

S1 Fig Quantification of *Ptbb-6*::*GFP* reporter protein induction following RNAi-mediated knockdown of mitochondrial ETC targets. All ETC subunits targeted by feeding RNAi in the current analysis—subunits are organized by complex (see S2 Table for a list of all known ETC subunits in *C*. *elegans*). Graphed data shows change in GFP fluorescence (mean +/-SD) following treatment of *Ptbb-6*::*GFP* reporter worms with each feeding RNAi. Data is normalized relative to vector-control treated worms. Each condition is the average fluorescence of between 3–12 worms. Asterisks indicate significantly different from vector *(Student’s t-test*, *p<0*.*05 before Sidak-Bonferroni correction for multiple testing)*. Red daggers indicate subunits with paralogs.
(TIF)

S2 Fig Quantification of *Pgst-4*::*GFP* reporter protein induction following RNAi-mediated knockdown of mitochondrial ETC targets. All ETC subunits targeted by feeding RNAi in the current analysis—subunits are organized by complex (see S2 Table for a list of all known ETC subunits in *C*. *elegans*). Graphed data shows change in GFP fluorescence (mean +/-SD) following treatment of *Pgst-4*::*GFP* reporter worms with each feeding RNAi. Data is normalized relative to vector-control treated worms. Each condition is the average fluorescence of between 3–12 worms. Asterisks indicate significantly different from vector *(Student’s t-test*, *p<0*.*05 before Sidak-Bonferroni correction for multiple testing)*. Red daggers indicate subunits with paralogs.
(TIF)

S3 Fig Quantification of *Phsp-6*::*GFP* reporter protein induction following RNAi-mediated knockdown of mitochondrial ETC targets. All ETC subunits targeted by feeding RNAi in the current analysis—subunits are organized by complex (see S2 Table for a list of all known ETC subunits in *C*. *elegans*). Graphed data shows change in GFP fluorescence (mean +/-SD) following treatment of *Phsp-6*::*GFP* reporter worms with each feeding RNAi. Data is normalized relative to vector-control treated worms. Each condition is the average fluorescence of between 3–12 worms. Asterisks indicate significantly different from vector *(Student’s t-test*, *p<0*.*05 before Sidak-Bonferroni correction for multiple testing)*. Red daggers indicate subunits with paralogs.
(TIF)

S4 Fig Presence of paralogs does not account for the heterogeneous expression of GFP reporter constructs following RNAi-mediated knockdown of subunits within each ETC complex. Data presented in Fig 2B of main text is reproduced on left. All ETC subunits with paralogs (marked with red daggers in S1–S3 Figs) were removed from the initial analysis and then GFP fluorescence re-averaged across each complex (+/-SD). Asterisks indicate significantly different from vector *(Student’s t-test*, *p<0*.*05 before Sidak-Bonferroni correction for multiple testing*,* p<0.*01*, **p<0.*001*, ***p<0.*0001)*.
(TIF)

S5 Fig RNAi-mediated disruption of non-ETC mitochondrial targets induce *Ptbb-6*::*GFP* reporter protein expression and long-life. RNAi clones targeting non-ETC mitochondrial targets and which have previously have been reported to increase lifespan also induce *Ptbb-6*::*GFP* expression. Targets include the mitochondrial ribosome machinery (B0261.4/*mrpl-47* [12] and *mrps-5* [122]); the solute carrier protein F13G3.7 [12] and the UPR^mt^ response protein *hsp-6* [123].
(TIF)

S6 Fig Quantification of *Ptbb-6*::*GFP*, *Pgst-4*::*GFP* and *Phsp-6*::*GFP* reporter expression in wild-type, *isp-1(qm150)*, *nuo-6(qm200)*, *ctb-1(qm189)* and *isp-1(qm150); ctb-1(qm189)* mutant backgrounds. (**A**) Constitutive GFP fluorescence in wild-type, *isp-1(qm150)*, *nuo-6(qm200)*, *ctb-1(qm189)* and *isp-1(qm150); ctb-1(qm189)* mutants carrying the listed reporter construct was averaged over 8–50 adult worms. Data is presented as relative GFP fluorescence (mean +/-SD). Two sets of statistical comparisons were undertaken: Asterisks indicate significant difference relative to wild-type control while hash indicates significant difference between *ctb-1(qm189)*) and *ctb-1(qm189); isp-1(qm150) (Student’s t-test*, *p<0*.*05 before Bonferroni correction for multiple testing*,* p<0.*017*, **p<0.*001*, ***p<0.*0001*, *# p<0*.*05*, *##p<0*.*002)*. (**B**) Wild-type worms and *ctb-1(qm189)* worms containing the listed GFP reporter construct were cultured on bacterial feeding RNAi targeting *atp-3 (1/10*^*th*^ *strength)*, *cco-1*, *isp-1* or *nuo-2* and GFP fluorescence quantified as described. Asterisks indicate significantly different relative to vector control of the same genetic background *(Student’s t-test*, *p<0*.*05 before Bonferroni correction for multiple testing*, *p<0.*01*, **p<0.*001*, ***p<0.*0001)*.
(TIF)

S7 Fig *Ptbb-6*::*GFP* reporter expression defines a UPR^mt^ independent pathway. (**A**) *isp-1(qm150)* worms carrying *Ptbb-6*::*GFP*, *Pgst-4*::*GFP* or *Phsp-6*::*GFP* reporter genes were exposed to *atfs-1* or *skn-1* feeding RNAi then GFP fluorescence was quantified in day one adults. Data is presented as mean (+/- SD) normalized to vector-control treated animals. Asterisks indicate significant difference relative to vector control-treated worms (*Student’s t-test*, *p<0*.*01; n* = 12 worms/condition, from four biological replicates). (**B**) RNAi knockdown of *skn-1* in *nuo-6(qm200)* worms turns off *Pgst-4*::*GFP*, as reported [59], but has no effect on *Ptbb-6*::*GFP* nor *Phsp-6*::*GFP* expression. RNAi knockdown of *atfs-1* blocks *Phsp-6*::*GFP* expression, as reported [43], but dramatically further upregulates *Ptbb-6*::*GFP*. Surprisingly, *atfs-1* RNAi also turned off *Pgst-4*::*GFP*. (**C**) *isp-1(qm150)*, *nuo-6(qm200)* and wild type worms containing the *Ptbb-6*::*GFP* reporter were cultured on RNAi to *atfs-1* and GFP fluorescence quantified as described in (A). Asterisks indicate significantly different relative to vector control-treated worms (*Student’s t-test*, *unequal variance*, *ns = not significant*,*p<0.*001*, **p<0.*0001)*.
(TIF)

S8 Fig Quantification of GFP reporter protein expression in *isp-1(qm150)* worms following RNAi-mediated knockdown of genes that function epistatically to *atfs-1* in the UPR^mt^. *isp-1(qm150)* worms containing *Ptbb-6*::*GFP*, *Pgst-4*::*GFP* or *Phsp-6*::*GFP* were cultured on feeding RNAi targeting components of the cellular surveillance pathway known to function upstream of *atfs-1* [26, 60]. GFP fluorescence was quantified when vector-control worms reached adulthood. Size-corrected fluorescence data is presented as mean fluorescence (+/- SD) normalized to vector-control treated animals. Asterisks indicate significant difference relative to vector control-treated worms (*Student’s t-test*, *p<0*.*001; n* = 6 worms/condition, from two biological replicates).
(TIF)

S9 Fig *Ptbb-6*::*GFP* retrograde pathway activation is under *pmk-3* MAPK control. (**A**) Induction of *Ptbb-6*::*GFP* expression in *isp-1(qm150);ctb-1(qm189)* worms is blocked by *pmk-3*, *sek-3* and *dlk-1* RNAi. Both *atfs-1* and *vhp-1* RNAi result in increased reporter fluorescence. Data is presented as mean (+/- SD) normalized to vector-control treated animals. Asterisks indicate significant difference relative to vector control-treated worms (*Student’s t-test*, *Bonferroni corrected for multiple testing p<0*.*01; n* = 5 worms/condition, from a single biological replicate). (**B**) The weak induction of *Ptbb-6*::*GFP* in *nuo-6(qm200)* worms is blocked when animals are exposed to *dlk-1*, *sek-3* or *pmk-3* RNAi, but none of these treatments have any effect on *Pgst-4*::*GFP* or *Phsp-6*::*GFP* reporter expression. (**C**) Wild type worms co-treated with RNAi targeting *pmk-3* and either *atp-3* or *isp-1* (both at 1/10^th^ strength) are unable to induce *Ptbb-6*::*GFP* expression.
(TIF)

S10 Fig (**A**) *nuo-6(qm200); Ptbb-6GFP* worms arrest growth when cultured on *vhp-1* RNAi (*top row*). Larval arrest is by-passed following the genetic removal of *pmk-3* in *pmk-3(ok169); nuo-6(qm200);Ptbb-6*::*GFP* worms (*bottom row*). (**B**) Knockdown of *pmk-3* by bacterial feeding RNAi uniquely rescued both the larval arrest and blocked *Ptbb-6*::*GFP* reporter expression of *isp-1(qm150); Ptbb-6*::*GFP* worms co-cultured on *vhp-1* RNAi (*top panel*, data copied from Fig 7, main text). Unlike *pmk-3* knockdown, *cbp-3* knockdown does not overcome the growth arrest induced by *vhp-1* knockdown.
(TIF)

S11 Fig Effect of MAPK RNAi, and *vhp-1*:*MAPK* combination RNAi (50:50), on wild-type worms containing the *Ptbb-6*::*GFP* reporter. (**A**) RNAi-mediated knockdown of *vhp-1* in *Ptbb-6*::*GFP* worms results in weak hypodermal GFP fluorescence and smaller adult worms. The decrease in adult size is proportional to *vhp-1* RNAi dose. (**B-D**) RNAi-mediated knockdown of none of the 14 known MAPKs in *C*. *elegans* inadvertently increases adult size.
(TIF)

S1 Table Genes upregulated independent of *atfs-1* upon *spg-7* RNAi. List of *C*. *elegans* genes up-regulated independently of *atfs-1* following exposure to *spg-7* RNAi. Shown are gene descriptions and fold induction in the presence and absence of *atfs-1* relative to vector control.
(XLSX)

S2 Table Effect of mitochondrial disruption on reporter gene expression in wild-type animals. *Ptbb-6*::*GFP*, *Pgst-4*::*GFP and Phsp-6*::*GFP* transcriptional reporter expression in wild-type worms following RNAi-mediated disruption of nuclear-encoded mitochondrial proteins.
(XLSX)

S3 Table Effect of mitochondrial disruption on reporter gene expression and development in *isp-1(qm150)* Mit mutants. *Ptbb-6*::*GFP*, *Pgst-4*::*GFP and Phsp-6*::*GFP* transcriptional reporter expression and development in *isp-1(qm150)* worms following RNAi-mediated disruption of nuclear-encoded mitochondrial proteins.
(XLSX)

S4 Table Lifespan statistics and Cox hazard analyses. Survival analyses (Excel sheet #1), and Genotype:RNAi interaction terms (Excel sheet #2).
(XLSX)

S5 Table Raw survival data. Raw survival data for the lifespan analyses used to generate S4 Table.
(XLSX)

S6 Table Worm strains. List of strains employed in current study, along with a description of relevant strain constructions.
(XLSX)

S1 Text Supplemental experimental procedures. Detailed list of experimental protocols.
(DOCX)
