# Supplementary figures and images for: DLK-1, SEK-3 and PMK-3 Are Required for the Life Extension Induced by Mitochondrial Bioenergetic Disruption in C. elegans
Source: PLoS Genet. 2016 Jul 15;12(7):e1006133. doi: 10.1371/journal.pgen.1006133 (PMC4946786; doi:10.1371/journal.pgen.1006133)

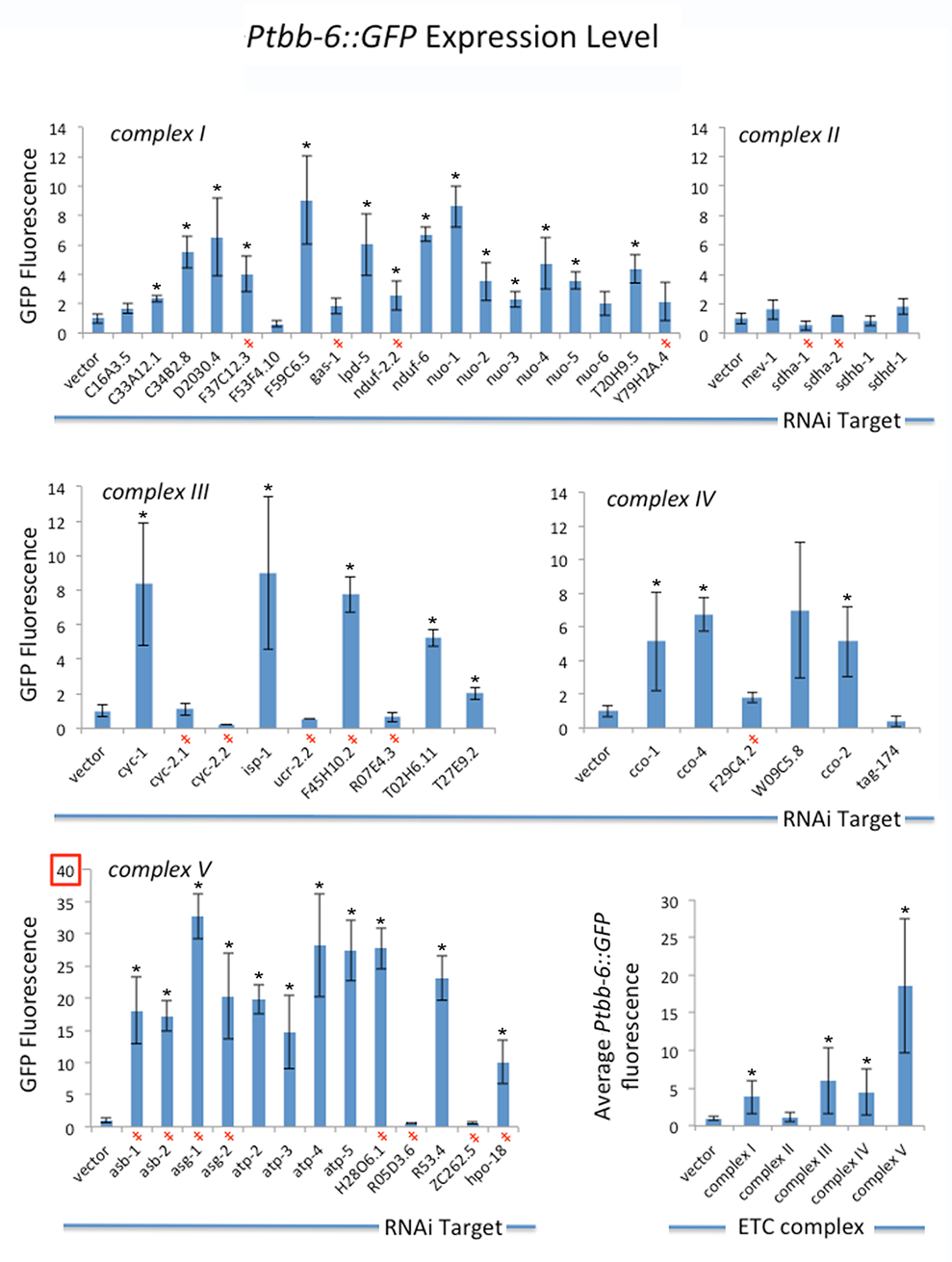

Supplement: S1 Fig — All ETC subunits targeted by feeding RNAi in the current analysis—subunits are organized by complex (see S2 Table for a list of all known ETC subunits in C. elegans). Graphed data shows change in GFP fluorescence (mean +/-SD) following treatment of Ptbb-6::GFP reporter worms with each feeding RNAi. Data is normalized relative to vector-control treated worms. Each condition is the average fluorescence of between 3–12 worms. Asterisks indicate significantly different from vector (Student’s t-test, p<0.05 before Sidak-Bonferroni correction for multiple testing). Red daggers indicate subunits with paralogs. (TIF) [file pgen.1006133.s001.tif]

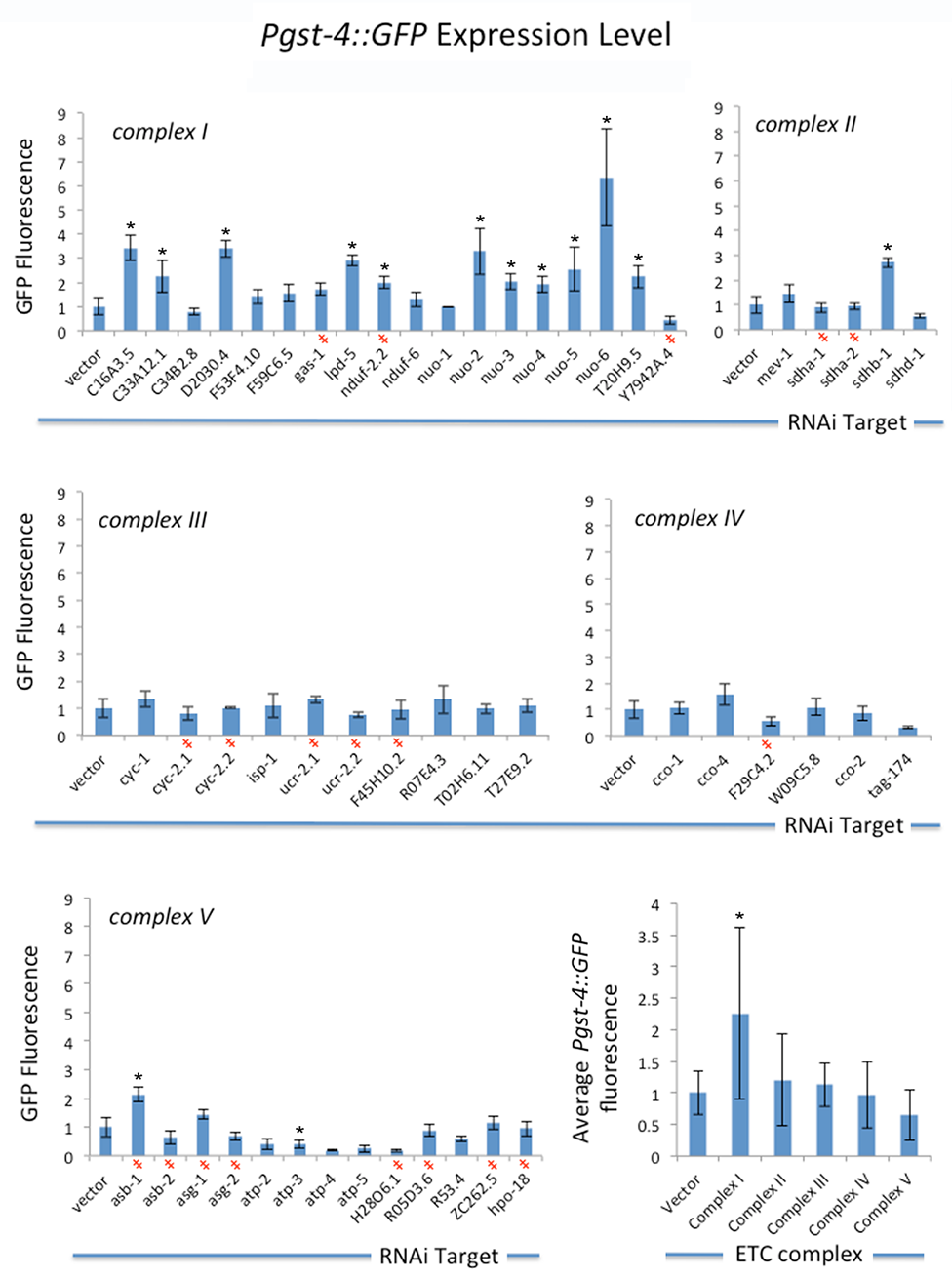

Supplement: S2 Fig — All ETC subunits targeted by feeding RNAi in the current analysis—subunits are organized by complex (see S2 Table for a list of all known ETC subunits in C. elegans). Graphed data shows change in GFP fluorescence (mean +/-SD) following treatment of Pgst-4::GFP reporter worms with each feeding RNAi. Data is normalized relative to vector-control treated worms. Each condition is the average fluorescence of between 3–12 worms. Asterisks indicate significantly different from vector (Student’s t-test, p<0.05 before Sidak-Bonferroni correction for multiple testing). Red daggers indicate subunits with paralogs. (TIF) [file pgen.1006133.s002.tif]

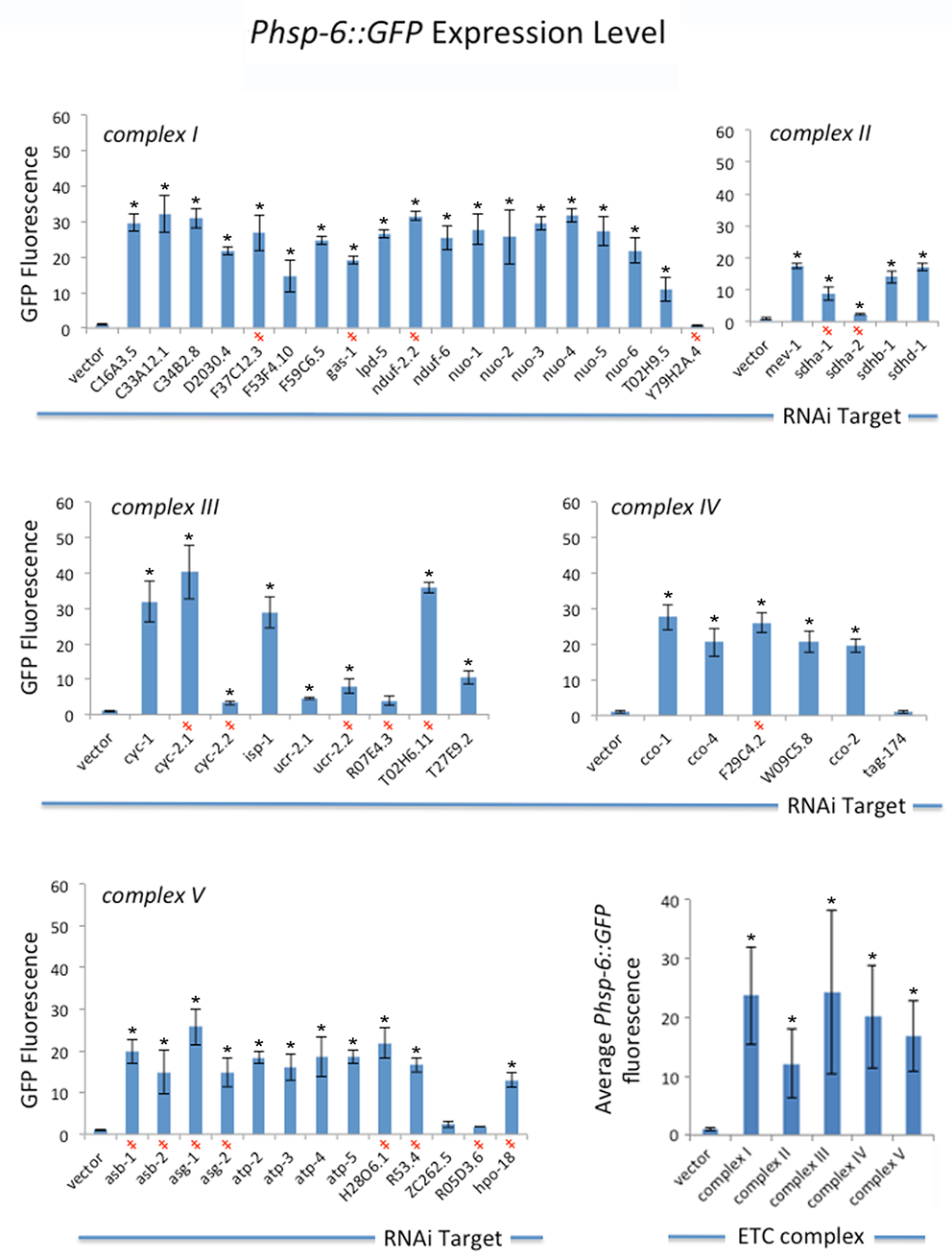

Supplement: S3 Fig — All ETC subunits targeted by feeding RNAi in the current analysis—subunits are organized by complex (see S2 Table for a list of all known ETC subunits in C. elegans). Graphed data shows change in GFP fluorescence (mean +/-SD) following treatment of Phsp-6::GFP reporter worms with each feeding RNAi. Data is normalized relative to vector-control treated worms. Each condition is the average fluorescence of between 3–12 worms. Asterisks indicate significantly different from vector (Student’s t-test, p<0.05 before Sidak-Bonferroni correction for multiple testing). Red daggers indicate subunits with paralogs. (TIF) [file pgen.1006133.s003.tif]

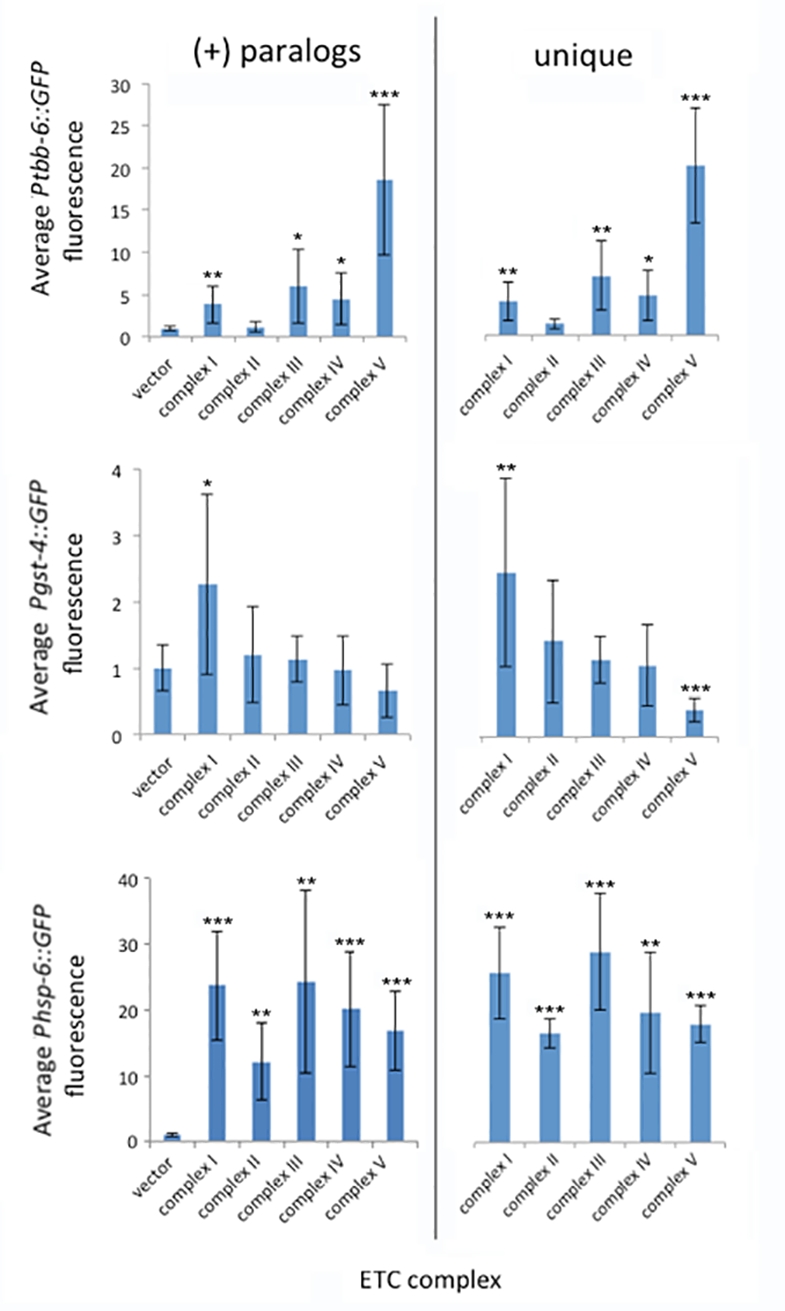

Supplement: S4 Fig — Data presented in Fig 2B of main text is reproduced on left. All ETC subunits with paralogs (marked with red daggers in S1–S3 Figs) were removed from the initial analysis and then GFP fluorescence re-averaged across each complex (+/-SD). Asterisks indicate significantly different from vector (Student’s t-test, p<0.05 before Sidak-Bonferroni correction for multiple testing,* p<0.01, **p<0.001, ***p<0.0001). (TIF) [file pgen.1006133.s004.tif]

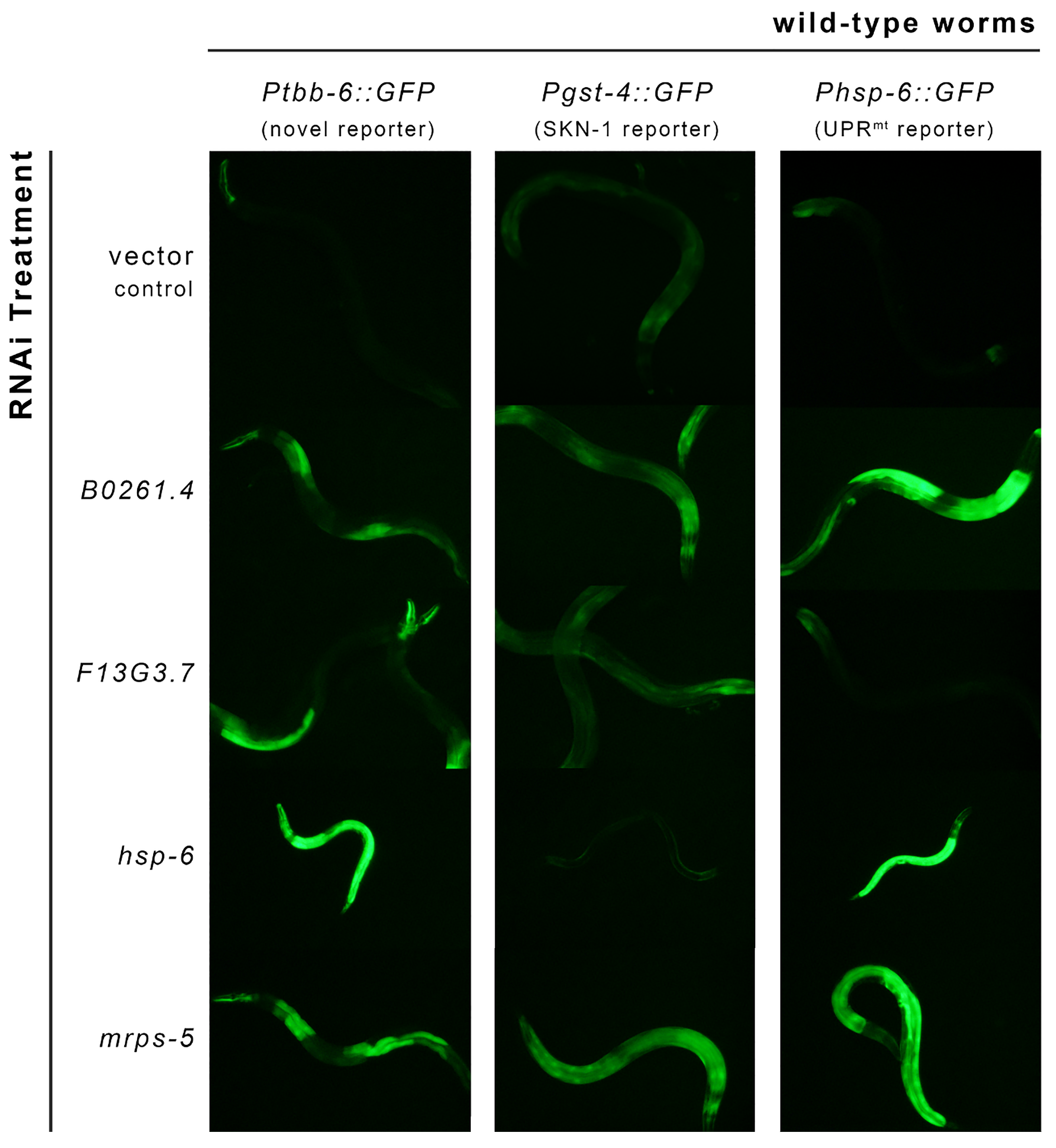

Supplement: S5 Fig — RNAi clones targeting non-ETC mitochondrial targets and which have previously have been reported to increase lifespan also induce Ptbb-6::GFP expression. Targets include the mitochondrial ribosome machinery (B0261.4/mrpl-47 [12] and mrps-5 [122]); the solute carrier protein F13G3.7 [12] and the UPRmt response protein hsp-6 [123]. (TIF) [file pgen.1006133.s005.tif]

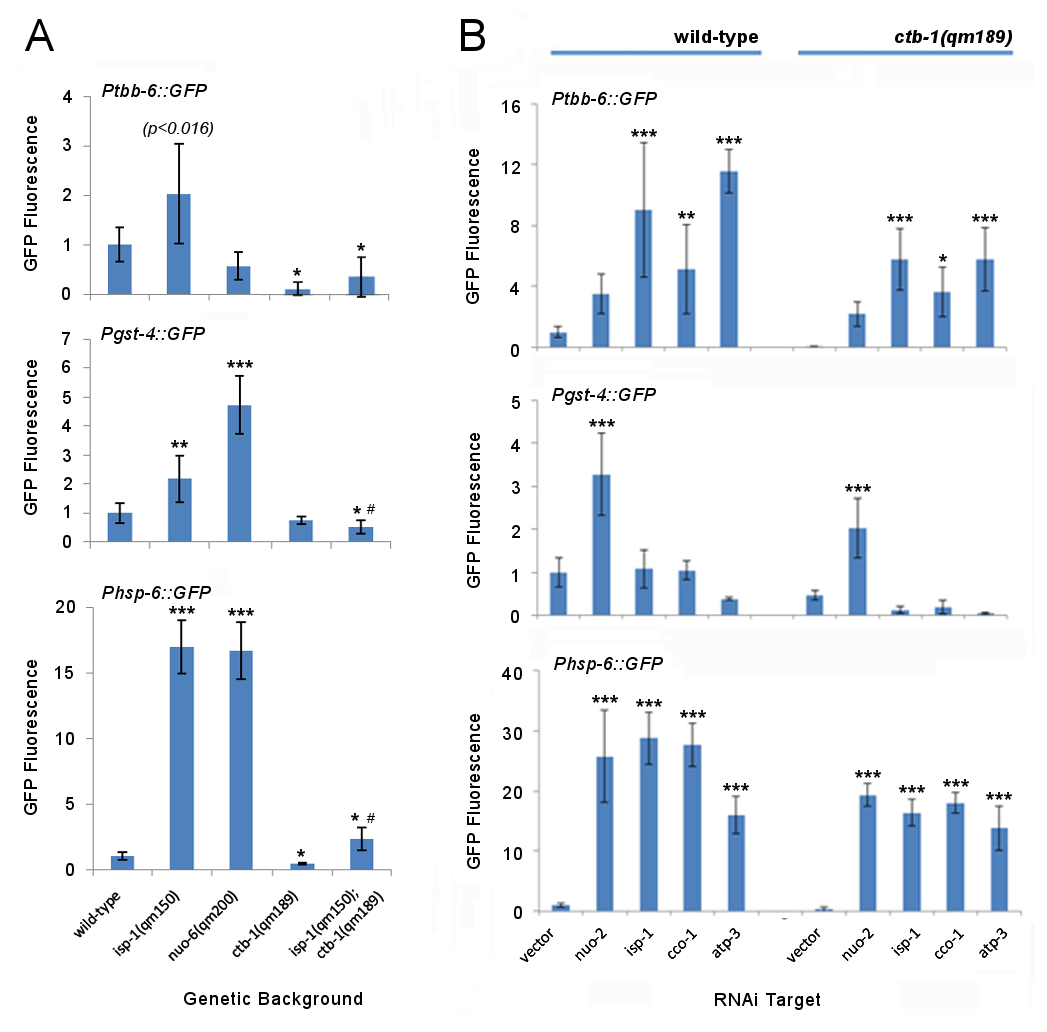

Supplement: S6 Fig — (A) Constitutive GFP fluorescence in wild-type, isp-1(qm150), nuo-6(qm200), ctb-1(qm189) and isp-1(qm150); ctb-1(qm189) mutants carrying the listed reporter construct was averaged over 8–50 adult worms. Data is presented as relative GFP fluorescence (mean +/-SD). Two sets of statistical comparisons were undertaken: Asterisks indicate significant difference relative to wild-type control while hash indicates significant difference between ctb-1(qm189)) and ctb-1(qm189); isp-1(qm150) (Student’s t-test, p<0.05 before Bonferroni correction for multiple testing,* p<0.017, **p<0.001, ***p<0.0001, # p<0.05, ##p<0.002). (B) Wild-type worms and ctb-1(qm189) worms containing the listed GFP reporter construct were cultured on bacterial feeding RNAi targeting atp-3 (1/10th strength), cco-1, isp-1 or nuo-2 and GFP fluorescence quantified as described. Asterisks indicate significantly different relative to vector control of the same genetic background (Student’s t-test, p<0.05 before Bonferroni correction for multiple testing, *p<0.01, **p<0.001, ***p<0.0001). (TIF) [file pgen.1006133.s006.tif]

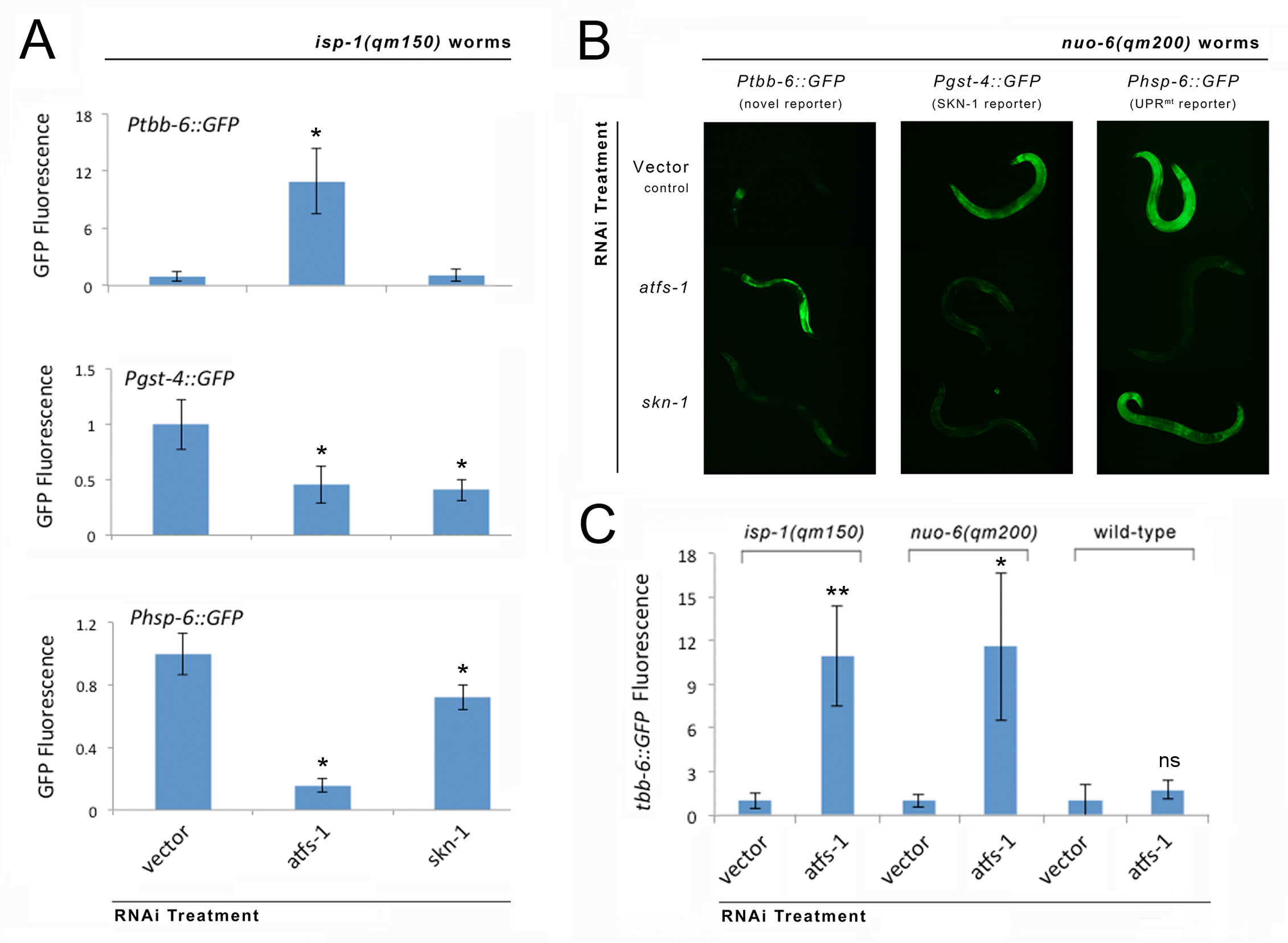

Supplement: S7 Fig — (A) isp-1(qm150) worms carrying Ptbb-6::GFP, Pgst-4::GFP or Phsp-6::GFP reporter genes were exposed to atfs-1 or skn-1 feeding RNAi then GFP fluorescence was quantified in day one adults. Data is presented as mean (+/- SD) normalized to vector-control treated animals. Asterisks indicate significant difference relative to vector control-treated worms (Student’s t-test, p<0.01; n = 12 worms/condition, from four biological replicates). (B) RNAi knockdown of skn-1 in nuo-6(qm200) worms turns off Pgst-4::GFP, as reported [59], but has no effect on Ptbb-6::GFP nor Phsp-6::GFP expression. RNAi knockdown of atfs-1 blocks Phsp-6::GFP expression, as reported [43], but dramatically further upregulates Ptbb-6::GFP. Surprisingly, atfs-1 RNAi also turned off Pgst-4::GFP. (C) isp-1(qm150), nuo-6(qm200) and wild type worms containing the Ptbb-6::GFP reporter were cultured on RNAi to atfs-1 and GFP fluorescence quantified as described in (A). Asterisks indicate significantly different relative to vector control-treated worms (Student’s t-test, unequal variance, ns = not significant,*p<0.001, **p<0.0001). (TIF) [file pgen.1006133.s007.tif]

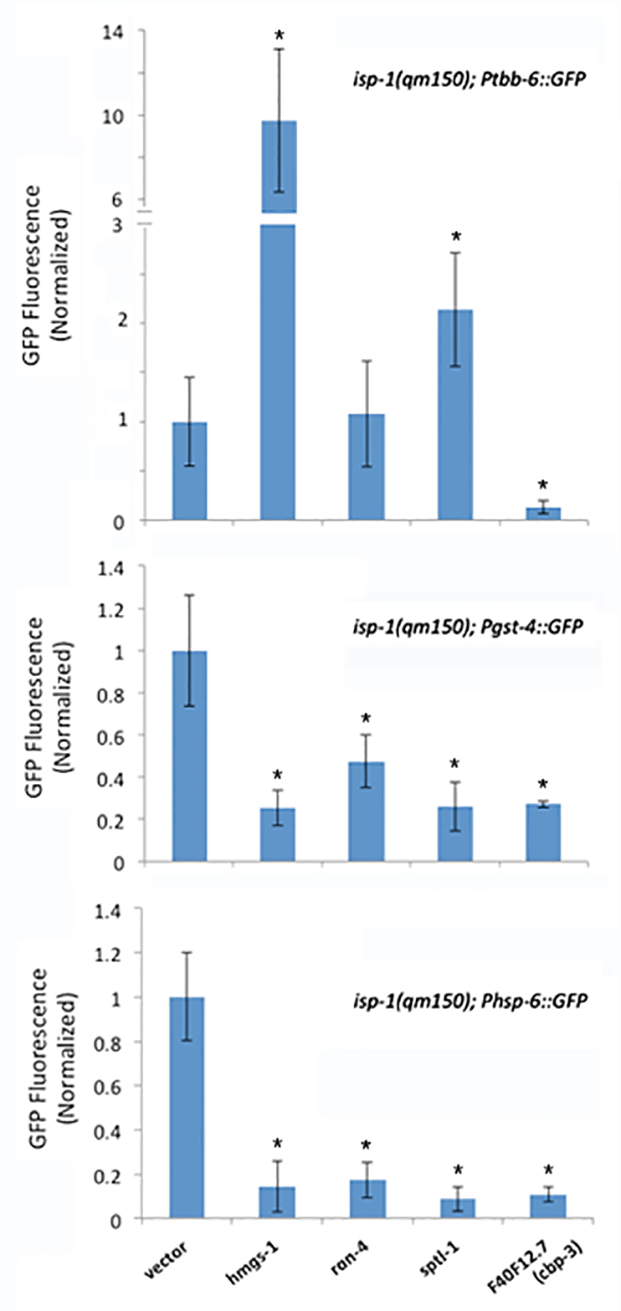

Supplement: S8 Fig — isp-1(qm150) worms containing Ptbb-6::GFP, Pgst-4::GFP or Phsp-6::GFP were cultured on feeding RNAi targeting components of the cellular surveillance pathway known to function upstream of atfs-1 [26, 60]. GFP fluorescence was quantified when vector-control worms reached adulthood. Size-corrected fluorescence data is presented as mean fluorescence (+/- SD) normalized to vector-control treated animals. Asterisks indicate significant difference relative to vector control-treated worms (Student’s t-test, p<0.001; n = 6 worms/condition, from two biological replicates). (TIF) [file pgen.1006133.s008.tif]

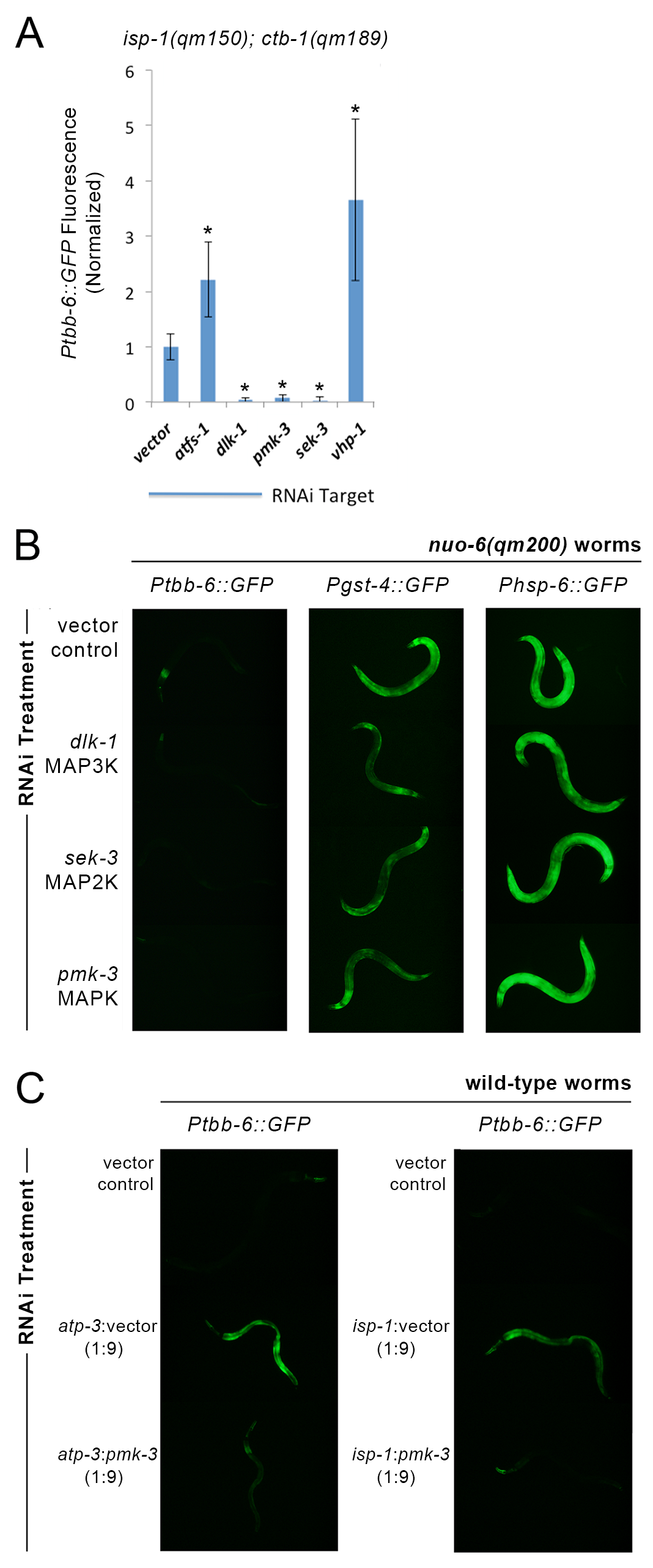

Supplement: S9 Fig — (A) Induction of Ptbb-6::GFP expression in isp-1(qm150);ctb-1(qm189) worms is blocked by pmk-3, sek-3 and dlk-1 RNAi. Both atfs-1 and vhp-1 RNAi result in increased reporter fluorescence. Data is presented as mean (+/- SD) normalized to vector-control treated animals. Asterisks indicate significant difference relative to vector control-treated worms (Student’s t-test, Bonferroni corrected for multiple testing p<0.01; n = 5 worms/condition, from a single biological replicate). (B) The weak induction of Ptbb-6::GFP in nuo-6(qm200) worms is blocked when animals are exposed to dlk-1, sek-3 or pmk-3 RNAi, but none of these treatments have any effect on Pgst-4::GFP or Phsp-6::GFP reporter expression. (C) Wild type worms co-treated with RNAi targeting pmk-3 and either atp-3 or isp-1 (both at 1/10th strength) are unable to induce Ptbb-6::GFP expression. (TIF) [file pgen.1006133.s009.tif]

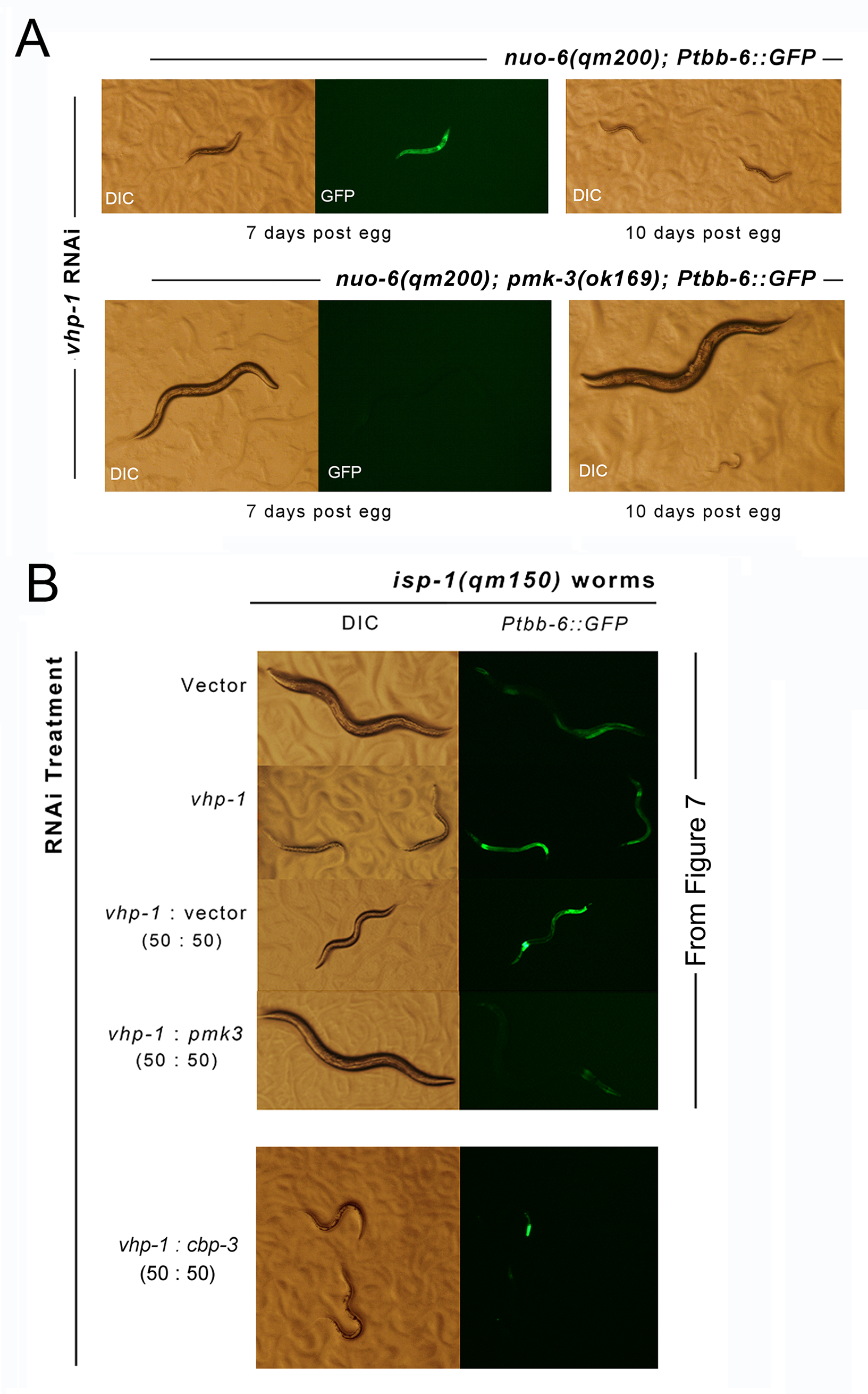

Supplement: S10 Fig — (A) nuo-6(qm200); Ptbb-6GFP worms arrest growth when cultured on vhp-1 RNAi (top row). Larval arrest is by-passed following the genetic removal of pmk-3 in pmk-3(ok169); nuo-6(qm200);Ptbb-6::GFP worms (bottom row). (B) Knockdown of pmk-3 by bacterial feeding RNAi uniquely rescued both the larval arrest and blocked Ptbb-6::GFP reporter expression of isp-1(qm150); Ptbb-6::GFP worms co-cultured on vhp-1 RNAi (top panel, data copied from Fig 7, main text). Unlike pmk-3 knockdown, cbp-3 knockdown does not overcome the growth arrest induced by vhp-1 knockdown. (TIF) [file pgen.1006133.s010.tif]

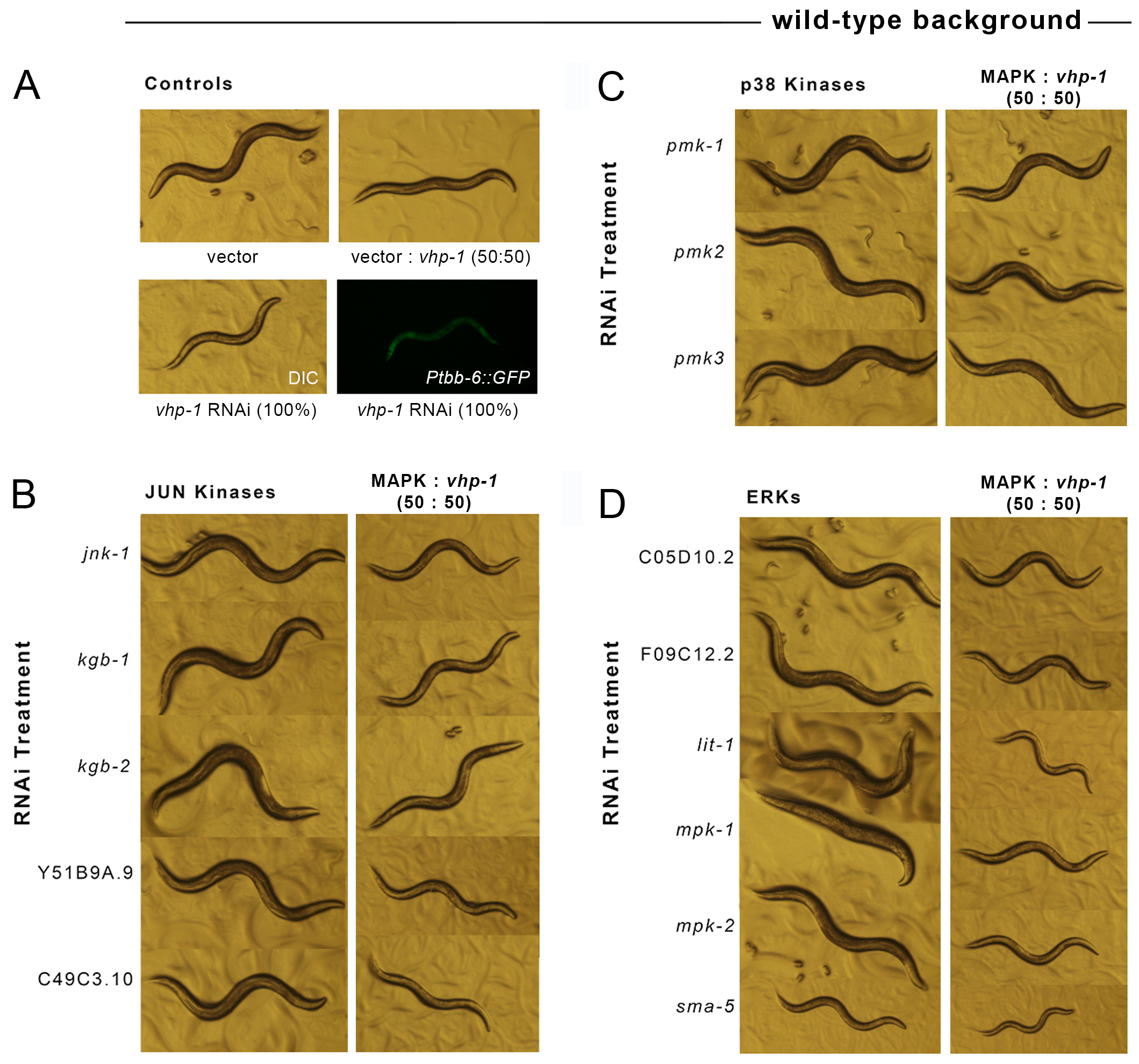

Supplement: S11 Fig — (A) RNAi-mediated knockdown of vhp-1 in Ptbb-6::GFP worms results in weak hypodermal GFP fluorescence and smaller adult worms. The decrease in adult size is proportional to vhp-1 RNAi dose. (B-D) RNAi-mediated knockdown of none of the 14 known MAPKs in C. elegans inadvertently increases adult size. (TIF) [file pgen.1006133.s011.tif]
