# Supplementary material for: DLK-1, SEK-3 and PMK-3 Are Required for the Life Extension Induced by Mitochondrial Bioenergetic Disruption in C. elegans
Source: PLoS Genet. 2016 Jul 15;12(7):e1006133. doi: 10.1371/journal.pgen.1006133 (PMC4946786; doi:10.1371/journal.pgen.1006133)
Supplement: S1 Text — Detailed list of experimental protocols. (DOCX) [file pgen.1006133.s018.docx]

**S1 Text: SUPPLEMENTAL EXPERIMENTAL PROCEDURES**

**Identification of *atfs-1* Independent Gene Set.** For this study, we utilized previously published microarray data (GEO#: GSE38196) of Haynes and colleagues [1]. mRNA for those studies was isolated from wild type and *atfs-1(tm4525)* worms that had been synchronized by bleach treatment, raised in liquid culture in the presence of *spg-7* or vector (pL4440) RNAi, then harvested at the L4 stage. mRNA was quantified using the GeneChip *C. elegans* genome array (Affymetrix). We combined the filtered data from each of three biological replicates and discarded all genes that were downregulated in wild type worms following *spg-7* RNAi treatment (*spg-7* RNAi/ vector ratio < 1). We did this because, later, when looking for genes upregulated in *atfs-1(tm4525)* mutants treated with *spg-7* RNAi, we wished to avoid selecting genes that were upregulated because ATFS-1 repression was relieved, as opposed to genes that were truly upregulated independently of ATFS-1. Next, we selected all genes upregulated 1.5 fold or higher relative to vector in wild type worms treated with *spg-7* RNAi, and which remained upregulated at a level of at least 1.8 fold or higher in mutant *atfs-1(tm4525)* worms following exposure to *spg-7* RNAi. Finally, from this set of genes we defined *atfs-1* independence as those genes that maintained an elevated level of expression on *spg-7* RNAi at 91% or higher of their expression value when ATFS-1 was present. This resulted in a list of 211 Affymetrix gene entries. The top 159 gene entries (148 unique genes) from this set were upregulated > 2 fold versus vector control-treated *atfs-1(tm4525)* worms and so we focused our attention on these genes in the following promoter analysis studies.

**Promoter Analyses.** Using the program WormMart [2], we downloaded 400 bp of the most proximal 5’ promoter sequence for each of the 148 genes described above. To identify conserved promoter elements in *atfs-1* independent genes, we employed the MEME Suite of tools (v4.10.1) [3, 4]. MAST [5], was used to locate DAF-16 binding sites using a weighted matrix based on the consensus motif identified by Kumar and colleagues [6].

ACGT alphabet frequency matrix

0.200000 0.150000 0.650000 0.000000

0.100000 0.000000 0.010000 0.890000

0.7500000 0.25000 0.000000 0.000000

0.980000 0.020000 0.000000 0.000000

0.750000 0.000000 0.250000 0.000000

0.000000 0.800000 0.050000 0.150000

0.800000 0.000000 0.000000 0.200000

**Nematode Strains and Maintenance**. **All strains used in this study are listed in Table S6.** Strains were maintained at 20ºC on standard NGM-Agar plates [7]. PCR was used to confirm the authenticity of the following strains: BS3383 [*pmk-3(ok169) IV*], RB1219 [*sek-3(ok1276) X*] and all lines containing the mitochondrial DNA-encoded *ctb-1(qm189)* mutation.

*ctb-1(qm189) seq fwd*: 5’—CGCCCGATAGGTTAATAGCAT—3’

*ctb-1(qm189) seq rev*: 5’—CACCGTGGCAATATAACCTAG—3’

*isp-1(qm150) seq fwd*: 5’—ACCAAGGCTGAGATTGCC—3’

*isp-1(qm150) seq rev*: 5’—CGTCCAGAAGCGTCGTAG—3’

*pmk-3(ok169) seq fwd*: 5’—GCTGACTCATATCTGATTGACCTC—3’

*pmk-3(ok169) seq rev*: 5’—GCTATGCGTGCGCGG—3’

*pmk-3 ext fwd:* 5’—TCGCCCTTTGTATGTCTTCC—3’

*pmk-3 int fwd*: 5’—TTTTCACTGCGTCTCAATCG—3’

*pmk-3 int rev*: 5’—TCAAATTTGCAGGTGTGCAGA—3’

*pmk-3 ext rev*: 5’—TTCTCCAGGGATTAACGGT—3’

*sek-3 inner fwd*: 5’—TTTCAACCACACGCCAAATA—3’

*sek-3 inner rev*: 5’—AAGTTGAGACGGACGAGGAA—3’

*sek-3 fwd* 2: 5’—CCCTGCACTGTTTCCAAGGACTTT—3’

*sek-3 fwd 3*: 5’—GGACGCAAACGGGCATACTTATGT—3’

*sek-3 fwd 4*: 5’—ATGTTTCAGGGTGTCGCCTGGAT—3’

*sek-3 fwd 5*: 5’—CCGTCCGTACATGGCTGTAAGTTT—3’

*sek-3 fwd 6*: 5’—CAATGGCGATGGATACGACA—3’

**Transgene Construction and Transgenic Strain Generation.**

*Ptbb-6::GFP* reporter construction: A 623 bp fragment comprising the entire upstream inter-gene region of *tbb-6* was PCR amplified from N2 Bristol (wild type), cloned into pPD95.77 in front of a GFP reporter open reading frame to form pCL179, and this plasmid was then introduced by microinjection into CL3162 [*smg-1(cc546)*]. The resulting multi-copy array was integrated via gamma irradiation and then the line was backcrossed several times to N2**.**

*Ptbb-6::mCherry* reporter construction: Assembly PCR using Phusion polymerase (NEB) was employed to connect a 596 bp proximal promoter fragment of *tbb-6* (genome coordinates: V:12261201- 12261797) with an mCherry ORF. The latter DNA fragment was derived from plasmid pGH8 (Addgene) and it also contained the *unc-54* 3’-UTR, separated from mCherry by an attB2 site. The resulting *Ptbb-6::mCherry::attB2::unc-54 3’UTR* fragment was blunt-end cloned into the Sma I site of pBluescript-SK(-). The resulting plasmid was then sequence verified. Primers used for assembly PCR were:

*Ptbb-6 fwd*: 5’—CGTTCATTCCAGTTGGACTAATAGTTTTAATACCGC—3’

*Ptbb-6 rev*: 5’—CACCCTTTGAGACCATTGTTCTCTGCAATTGAG—3’

*Ptbb-6::mCherry fwd*: 5’—CCCTCAATTGCAGAGAACAATGGTCTCAAAGGGTGAAG—3’

*Ptbb-6::mCherry rev*: 5’—AAGTTGGAAACAGTTATGTTTGGTATATTGGG—3’

*Ptbb-6::ΔCCAAT (A site)::GFP* reporter construction: Phusion/T4 ligase-based site-directed mutagenesis was used to delete the left most C/EBP-like binding site in the promoter of *tbb-6* (refer to Figure 1C for gene co-ordinates). pCL179 was used as the parent plasmid for mutagenesis along with the following two primers:

*fwd*: 5’—TAGACTATATTTCGCAATAGGGAATAGGTATTTAA—3’

*rev*: 5’—TTGATCTGCAAGAAAACTGCGGAA—3’

The resulting plasmid was co-injected into N2 along with *Ptbb-6::mCherry::attB2::unc-54 3’UTR* /pBluescript (each at a final concentration of 100 ng/μL, to generate line SLR0136.

*Ptbb-6::ΔCCAAT (B site)::GFP* reporter construction: The GeneArt Site-Directed Mutagenesis Plus kit (ThermoFisher CAT A14604) was used to delete the right most C/EBP-like binding site in the promoter of *tbb-6* (refer to Figure 1C for gene co-ordinates). pCL179 was used as the parent plasmid for mutagenesis along with the following two primers:

*fwd*: 5’— TCGCAATAGACTATATAGGGAATAGGTATT —3’

*rev*: 5’— AATACCTATTCCCTATATAGTCTATTGCGA —3’

The resulting plasmid was co-injected into N2 along with *Ptbb-6::mCherry::attB2::unc-54 3’UTR* /pBluescript (each at a final concentration of 100 ng/μL), to generate line SLR0137.

*Ptbb-6:: ΔΔCCAAT (A+B site)::GFP* reporter construction: Phusion/T4 ligase-based site-directed mutagenesis was used to delete both C/EBP-like binding sites in the promoter of *tbb-6* (refer to Figure 1C for gene co-ordinates). pCL179 was used as the parent plasmid for mutagenesis along with the following two primers:

*fwd*: 5’— TAGGGAATAGGTATTTAAAGAGAGG —3’

*rev*: 5’— TATAGTCTATTGATCTGCAAGAAAAC —3’

The resulting plasmid was co-injected into N2 along with *Ptbb-6::mCherry::attB2::unc-54 3’UTR* /pBluescript (each at a final concentration of 100 ng/μL), to generate line SLR0138.

*Constitutively-active, neuronal DLK-1* **:** Construction of plasmid *pCZGY#1969(Prgef-1::dlk-1 L(EE)); pRF4(rol-6(su1006)* has been described previously [8], and was graciously provided Dr. Yishi Jin (USCD). This construct contains a constitutively active form of DLK-1, containing dual phosphomimetic mutations in its activation domain, coupled to the neural-specific *rgef-1* promoter. pCZGY#1969 was microinjected at a concentration of 100 ng/μL into SLR0115 [*dvIs67[pCL179(Ptbb-6::GFP)*] worms to generate SLR0127 **[***dvIs67[pCL179(Ptbb-6::GFP)]; stxEx2[pCZGY#1969(Prgef-1::dlk-1 L(EE)); pRF4(rol-6(su1006))]***]** animals.

**Feeding RNAi.** Bacterial feeding RNAi clones targeting *cbp-3* (F40F12.7), *cco-2*, *jkk-1*, *mek-2*, *sdha-1*, *sma-5*, *tag-174*, T27E9.2, *vms-1*, Y54E10BR.5 and Y54F10BM.13 were constructed using the following primers:

*cbp-3* *fwd*: 5’—CCCGTGAAGCGGAAGCTTATTCAGC—3’

*cbp-3* *rev:* 5’—CCGGGCAATCTTCCCTCGAGCAGTCTTTCC-3’

cco-2 *fwd*: 5’—TCTCCTCGAGGCTGTAACCCGACTGGC—3’

cco-2 *rev*: 5’—ACACTGAGATCTCCGGCTCTGGCACG—3’

*jkk-1 fwd*: 5’—AAAAAGCTTATCTGCGACGGTTCGC—3’

*jkk-1 re*v: 5’—AAAACTCGAGCGCAAGCATAGGTCCAAGA—3’

*mek-2 fwd*: 5’—GTACACCTCGAGATCAAGCCG—3’

*mek-2 rev*: 5’—AAAAAAAGATCTGGGTTGCTCGTTCACTTGG—3’

*sdha-1* *fwd*: 5’—ACCAAGCTTACTTGCTGTGTTGCTGATCG—3’

*sdha-1* *rev*: 5’—GGGGCTCGAGAAGCTCGGCAGTTGAGATGT—3’

*sma-5* *fwd:* 5’—GACTCTAGATCTCCCTCTCCGTGACATTTTCCG—3’

*sma-5* *rev*: 5’—CATCTCCTCGAGCTTTGC—3’

*tag-174 fwd*: 5’—TTCACTCGAGCCGCTCGGTCGTCAAG—3’

*tag-174 rev*: 5’—TCTACGAGATCTGCTCCGCCTTGTTGTGG—3’

T27E9.2 *fwd*: 5’—GCCACTCGAGGCTGAC—3’

T27E9.2 r*ev*: 5’—TCTCTCAGATCTCGAAGCGAAGGCCTTTG—3’

*vms-1* *fwd*: 5’—CCCAAGCTTCCAAACAAGGAGGAGTCCAA—3’

*vms-1* r*ev*: 5’—GGAACTCGAGCAGTCTCATTTTTGACGCGA—3’

Y54E10BR.5 *fwd*: 5’—CGTCCTCGAGGCGATGTTCAGCGAGATCCGCCAG—3’

Y54E10BR.5 *rev*: 5’—CATAAGCTTGGACGAAGAGACCGAGGAAGGCG—3’

Y54F10BM.13 *fwd:* 5’—GTCCCCAGATCTCCCACAATTCCCTCATTCTG—3’

Y54F10BM.13 *rev:* 5’—CACACTCGAGCATATTTCTTGGAGAGCC—3’

Gene fragments were amplified from N2 genomic DNA (except for mek-2 and *Y54E10BR.5* which were both amplified from N2 cDNA), and cloned into pL4440. RNAi targeting *isp-1* has been described previously [7]. RNAi constructs targeting *atfs-1*, *jnk-1*, *kgb-1*, *pmk-1*, *pmk-2*, *zip-*3, C49C3.10, Y51B9A.9 were provided by Dr. Cole Haynes (Memorial Sloan Kettering Center). *sek-1* RNAi was provided by Dr. Danielle Garsin (UTHSC, Houston). All remaining RNAi constructs were derived from the Ahringer RNAi library [9]. Feeding RNAi and dilution was performed as described previously [7].

**Fluorescence Imaging and Quantification**. To assay GFP reporter induction, eggs or arrested L1 larvae were placed on control (pL4440 vector) and test RNAi lawns and then photographed as first-day gravid adults, except in the case of larval arrest. Since some RNAi result in a delay of development, plates were often photographed more than once over the course of several days. Multiple worms were imaged (from two to more than a dozen per condition) across independent experiments. All animals were imaged at the same developmental age (day one of adulthood), or at the same chronological age when appropriate. Images were captured using an Olympus DP71 CCD camera connected to an Olympus SZX16 fluorescence dissecting microscope. All images were quantified using ImageJ software (NIH), followed by statistical sample comparison using Prism 6.0 software. All comparisons were performed using the, Student’s t-test with significance set at p <0.05 (where applicable, data was corrected for multiple testing using either the Sidak-Bonferroni or Bonferroni algorithm). For comparison of multiple treatment groups in **Fig. 6A**, a one-way ANOVA was used and then a post-hoc analysis was performed using Dunnett’s test.

***atfs-1* mRNA Quantification.** Arrested L1 (2000/condition) from strains SLR115 *(Ptbb-6::GFP)*, SLR117 *(isp-1(qm150); Ptbb-6::GFP),* and SLR65 *(nuo-6(qm200); Ptbb-6::GFP)* were spotted onto vector (pL4440) and *atfs-1* RNAi plates. Worms were grown until day 1 of adulthood, then collected and washed with S-Basal media. Total RNA was extracted using a combination of Trizol/chloroform and an RNeasy kit (Qiagen). mRNA was amplified from 1 μg of total RNA using an oligo d(T) primer, and then cDNA generated using a High Capacity cDNA Reverse Transcription kit (Applied Biosystems). qPCR was performed using RT^2^ SYBR Green Mastermix (Qiagen), a Stratagene Mx3000P qPCR machine, and the following gene-specific primers:

*cdc-42* *fwd*: 5’—CTGCTGGACAGGAAGATTACG—3’

*cdc-42 rev:* 5’—CTCGGACATTCTCGAATGAAG—3’

*pmp-3 fwd*: 5’—GTTCCCGTGTTCATCACTCAT—3’

*pmp-3 rev:* 5’—ACACCGTCGAGAAGCTGTAGA—3’

Y45F10D.4 *fwd*: 5’—GTCGCTTCAAATCAGTTCAGC—3’

Y45F10D.4 *rev:* 5’—GTTCTTGTCAAGTGATCCGACA—3’

*atfs-1 fwd*: 5’—CCCTTTGTTCACCCAACTGT—3’

*atfs-1 rev:* 5’—TGGGATCGAATACCCGTAAC—3’

Primer sequences for *cdc-42, pmp-3,* and *y45F10D.4* were described in [10], and AmplifX was used to design the *atfs-1* primer pair. All primers were synthesized by Sigma-Aldrich (MO). Genes were normalized using the geometric mean of *cdc-42, pmp-3* and *y45F10D*.4 following the procedure of Hoogewijs et. al. [10]. Calculation of fold change in expression was done using the ΔΔC_t_ method. Results are averages of three biological replicates. Error bars represent standard deviation.

**Lifespan Analyses**. Lifespans were performed as described previously without the use of FudR and all plates were maintained at 20ºC [7]. The first day of adulthood was designated as day 1. Survival data was analyzed in R 3.0.2 with the survival package. We used a Log-Rank analysis to analyze the effect of RNAi-induced knockdown on a single strain and the Cox proportional-hazard model to ascertain significant strain:RNAi interactions. A full description of all lifespan experiments is provided in **Table S4.** Raw lifespan data is provided in Table **S5**. Graphs were created in R 3.0.2 with the ggplot2 package and code by Edwin Thoen posted on the R-statistics blog.

**SUPPLEMENTAL REFERENCES**

1. Nargund AM, Pellegrino MW, Fiorese CJ, Baker BM, Haynes CM. Mitochondrial import efficiency of ATFS-1 regulates mitochondrial UPR activation. Science. 2012;337(6094):587-90. Epub 2012/06/16. doi: 10.1126/science.1223560. PubMed PMID: 22700657.

2. Schwarz EM, Antoshechkin I, Bastiani C, Bieri T, Blasiar D, Canaran P, et al. WormBase: better software, richer content. Nucleic Acids Res. 2006;34(Database issue):D475-8. doi: 10.1093/nar/gkj061. PubMed PMID: 16381915; PubMed Central PMCID: PMC1347424.

3. Ma W, Noble WS, Bailey TL. Motif-based analysis of large nucleotide data sets using MEME-ChIP. Nat Protoc. 2014;9(6):1428-50. doi: 10.1038/nprot.2014.083. PubMed PMID: 24853928; PubMed Central PMCID: PMC4175909.

4. Bailey TL, Elkan C. Fitting a mixture model by expectation maximization to discover motifs in biopolymers. Proc Int Conf Intell Syst Mol Biol. 1994;2:28-36. PubMed PMID: 7584402.

5. Bailey TL, Gribskov M. Combining evidence using p-values: application to sequence homology searches. Bioinformatics. 1998;14(1):48-54. PubMed PMID: 9520501.

6. Kumar N, Jain V, Singh A, Jagtap U, Verma S, Mukhopadhyay A. Genome-wide endogenous DAF-16/FOXO recruitment dynamics during lowered insulin signalling in C. elegans. Oncotarget. 2015;6(39):41418-33. doi: 10.18632/oncotarget.6282. PubMed PMID: 26539642; PubMed Central PMCID: PMCPMC4747164.

7. Rea SL, Ventura N, Johnson TE. Relationship Between Mitochondrial Electron Transport Chain Dysfunction, Development, and Life Extension in Caenorhabditis elegans. PLoS Biol. 2007;5(10):e259. Epub 2007/10/05. doi: 06-PLBI-RA-2325 [pii]

10.1371/journal.pbio.0050259 [doi]. PubMed PMID: 17914900.

8. Yan D, Jin Y. Regulation of DLK-1 kinase activity by calcium-mediated dissociation from an inhibitory isoform. Neuron. 2012;76(3):534-48. Epub 2012/11/13. doi: 10.1016/j.neuron.2012.08.043. PubMed PMID: 23141066; PubMed Central PMCID: PMC3508676.

9. Kamath RS, Ahringer J. Genome-wide RNAi screening in Caenorhabditis elegans. Methods. 2003;30(4):313-21.

10. Hoogewijs D, Houthoofd K, Matthijssens F, Vandesompele J, Vanfleteren JR. Selection and validation of a set of reliable reference genes for quantitative sod gene expression analysis in C. elegans. BMC Mol Biol. 2008;9:9. PubMed PMID: 18211699.
